# PRMT5 promotes ferroptosis in cisplatin-induced acute kidney injury through WDR5/ACSL4 pathway

**DOI:** 10.1016/j.redox.2026.104421

**Published:** 2026-10-01

**Authors:** Jiawen Lin, Junxuan Fang, Tianjiao Cui, Jiaqi Xu, Wenjian Lin, Shuping Li, Yuan Sui, Zhihua Zheng, Keping Wu, Mingcheng Huang

**Affiliations:** a Department of Nephrology, Kidney and Urology Center, The Seventh Affiliated Hospital, Sun Yat-Sen University, Shenzhen, Guangdong, China; b Department of Physiology, University of Oklahoma Health Sciences Center, Oklahoma City, USA; c Molecular and Cellular Biology Laboratory, The Salk Institute for Biological Studies, La Jolla, CA, USA; d Department of Nephrology, Kidney and Urology Center, The Sixth Affiliated Hospital, Sun Yat-Sen University, Guangzhou, China

**Keywords:** Acute kidney injury (AKI), Cisplatin, Ferroptosis, Lipid peroxidation, Protein methyltransferase 5 (PRMT5)

## Abstract

The epigenetic mechanisms driving the transition to ferroptosis in acute kidney injury (AKI) remain unclear. In this study, protein methyltransferase 5 (PRMT5) and its partner MEP50 were significantly upregulated in the renal tubules of AKI patients and murine models. Genetic silencing of PRMT5 via AAV-shRNA in vivo and siRNA in vitro improved renal function, attenuated mitochondrial damage, and suppressed ferroptotic tubular cell death. Mechanistically, PRMT5 promotes ACSL4 expression in a WDR5-dependent manner, facilitating tubular ferroptosis. Pharmacological inhibition of PRMT5 (GSK591) post-treatment or cloperastine hydrochloride prophylactic administration similarly improved renal function and limited ferroptotic cell death. These findings offer novel insights into the upstream epigenetic regulation of ACSL4-driven ferroptosis and suggest that PRMT5-ACSL4 axis might be a potential therapeutic target for AKI intervention.

## Highlights

• Cisplatin (CP) induces PRMT5 nuclear accumulation in tubular epithelial cells.
• PRMT5 promotes lipid peroxidation and tubular ferroptosis in CP-AKI.
• The PRMT5/WDR5/ACSL4 axis activation induces ferroptotic tubules.
• Genetic or pharmacological inhibition of PRMT5 alleviates CP nephrotoxicity.
• PRMT5 is a potential therapeutic target for mitigating redox imbalance in CP-AKI.

## Graphical abstract

**Figure:**
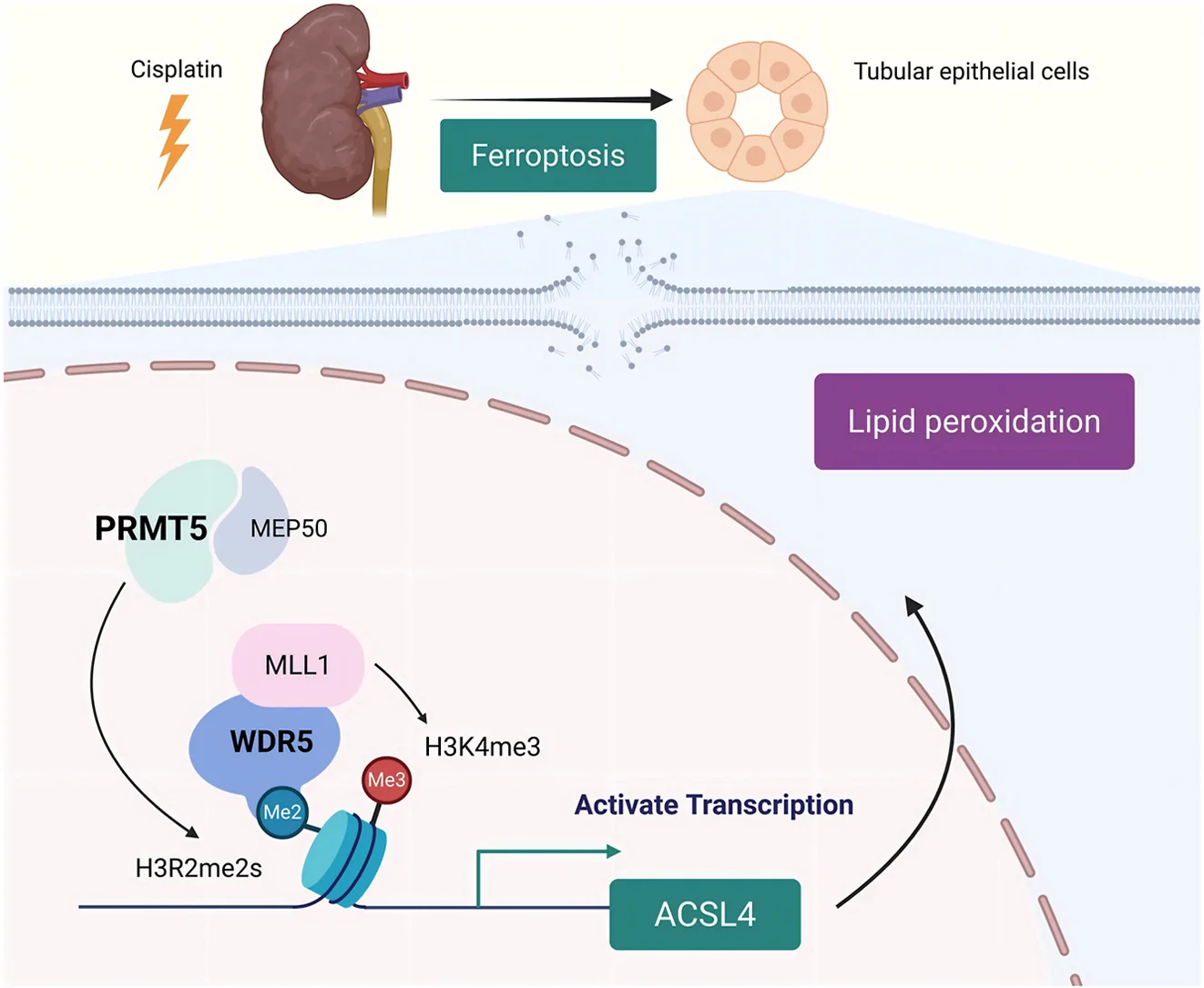

## 1 Introduction

Acute kidney injury (AKI) is a complex clinical syndrome defined by a rapid decline in glomerular filtration rate and disruption of electrolyte homeostasis [1]. Despite significant therapeutic advances, AKI remains a primary driver of global morbidity and mortality, with high rates of progression to chronic kidney disease (CKD) and end-stage renal disease (ESRD) [[2], [3], [4]]. While AKI etiologies are diverse, including ischemia-reperfusion injury and sepsis, cisplatin-induced acute kidney injury (CP-AKI) remains a major driver of iatrogenic renal injury, affecting approximately 30% of patients [5]. Renal proximal tubular epithelial cells (TECs) are the primary targets of cisplatin toxicity, a vulnerability dictated by their high metabolic demand and the preferential sequestration of the drug via organic cation transporters (OCTs) [6,7]. Intracellular cisplatin accumulation triggers mitochondrial dysfunction, leading to the excessive generation of reactive oxygen species (ROS) and a robust inflammatory response. These redox imbalances and pathological events culminate in extensive TEC loss, ultimately leading to renal failure [8,9]. Nowadays the molecular transition from oxidative stress to tubular death remains incompletely understood.

Ferroptosis is a regulated necrotic process driven by iron-dependent lipid peroxidation, and it contributes to the tubular cell loss in AKI [10,11]. This process involves two converging mechanisms: activation of lipid peroxidation and failure of antioxidant defense. Acyl-CoA synthetase long-chain family member 4 (ACSL4) is a critical executioner, enriching membrane phospholipid with polyunsaturated fatty acids (PUFAs) that serve as substrates for lipid hydroperoxide formation [[12], [13], [14]]. While the NRF2-GPX4 axis functions as the central antioxidant safeguard that limits ferroptotic damage [15,16]. Despite the identification of these metabolic checkpoints, upstream epigenetic mechanism that regulate the ferroptotic program in AKI remains poorly understood [17].

Protein arginine methyltransferase 5 (PRMT5) is the predominant Type II methyltransferase and a major epigenetic regulator that catalyzes the symmetric di-methylation of arginine (SDMA) residues on histone and non-histone proteins [18]. In complex with its cofactor methylosome protein 50 (MEP50), PRMT5 modulates transcriptional programs involved in metabolism [19], oxidative stress [20], and cell fate decisions [21]. Its function depends on genomic recruitment, allowing context-dependent activation or repression of target genes [22]. While PRMT5 has been implicated in regulating ferroptosis in cancer [[23], [24], [25], [26]], its role in AKI has not been explored.

In this study, PRMT5 was identified as an epigenetic driver of ferroptosis in AKI. PRMT5 was upregulated in renal tubules of AKI patients and murine models, correlating with tubular injury. Mechanistically, PRMT5 promoted ACSL4 driven lipid peroxidation through WDR5 dependent chromatin regulation. Genetic or pharmacological inhibition of PRMT5 reduced lipid oxidative damage, preserved tubular integrity, and alleviated AKI severity. These findings reveal PRMT5 as a potential therapeutic target in nephrotoxic kidney injury.

## 2 Materials and methods

### 2.1 Human kidney specimens

All procedures involving human samples were performed in compliance with ethical standards and were approved by the Ethics Committee (Approval No. KY-2026-015-01). Informed consent was obtained from all participants prior to sample collection. Renal biopsy samples were acquired from patients with AKI. Control tissues consisted of para-carcinoma renal specimens obtained from patient with renal carcinoma, which exhibited no histopathological evidence of tubular injury. The patients’ clinical information and parameters are presented in Supplementary Table 1.

### 2.2 Cell culture and treatment

HK-2 cells (human proximal tubular epithelial cells) at passage 5-10 were maintained in DMEM/F12 medium supplemented with 10% fetal bovine serum (FBS) and 1% penicillin-streptomycin at 37°C in 5% CO_2_ humidified atmosphere. To induce injury, cells were exposed to 10 μM or 20 μM cisplatin for 24 h. For gene knockdown, transfection was performed using PRMT5-targeted or WDR5-targeted siRNA (GenePharma) and transfection reagents (GenePharma) according to the manufacturer's protocol. For gene stable overexpression, HK-2 cells were transduced with PRMT5 overexpressing lentivirus (OE-PRMT5, GeneChem) and subsequently selected with 2 μg/mL puromycin for 5 days. The scramble lentivirus (OE-NC) and scrambled siRNA (siRNA-NC) were transfected as control. The siRNA and lentivirus sequencing are provided in Supplementary Table 2.

### 2.3 Animal models and treatment

All animal experiments were approved by the Institutional Animal Care and Use Committee (Approval No. SYSU-IACUC-2024-002652). Male C57BL/6 mice (6-8 weeks old, 18-22 g body weight) were obtained from GemPharmatech Corporation. Mice were housed under standard conditions and acclimatized for one week before the experiment.

Three distinct AKI models were established: (1) CP-AKI via a single intraperitoneal (i.p.) injection of 10 or 20 mg/kg cisplatin, with samples collected 72 h post injection; (2) LPS induced sepsis-associated AKI (S-AKI) via i.p. injection of 20 mg/kg lipopolysaccharide (LPS), with samples collected 24 h after injection; and (3) folic acid-induced AKI (FA-AKI) via i.p. injection of 250 mg/kg folic acid, with samples collected 48 h post-injection [27].

For tubular-specific adeno-associated virus 9 mediated PRMT5 knockdown, mice were anesthetized, and the left kidney was exposed. AAV9-GGT-shPRMT5 (AAV-shPRMT5, GeneChem, 10 μl of 1 × 10^11^ genome copies per mouse) was injected into the renal pelvis using a 33-gauge needle, while AAV9-GGT-scramble (AAV-NC) served as control. Mice were allowed to recover for 28 days to ensure stable transgene expression before CP-AKI modeling.

For pharmacological intervention studies, the following regimens were used: (1) the PRMT5-specific inhibitor GSK591 was i.p. injected at a dose of 50 mg/kg 1 h after cisplatin administration and daily for 3 days [28,29]; (2) cloperastine hydrochloride were i.p. injection at doses of 10 or 30 mg/kg once daily for 3 consecutive days prior to AKI induction [30,31]. At the designated endpoints, blood and kidney samples were collected for subsequent analysis. The summary of pharmacological agents and treatment regimens in animal experiments are listed in Supplementary Table 3. The shRNA sequences packaged in AAV9-shPRMT5 and the control construct details for AAV9-NC are provided in Supplementary Table 4, and reagents used are listed in Supplementary Table 5.

### 2.4 Assessment of renal function and histopathology

Renal function was assessed by measuring serum creatinine (SCr) and urea nitrogen (BUN) using an automatic biochemical analyzer. The kidney-to-body weight ratio was determined by excising whole kidneys and weighing them immediately. The ratio was calculated as (kidney wet weight/body weight) ×100%.

For histological analysis, kidney tissues were fixed in 4% paraformaldehyde, embedded in paraffin, and sectioned at 4 μm thickness. Hematoxylin and eosin (H&E) staining and Periodic acid-Schiff (PAS) staining was performed for morphometric analysis. Renal tubular injury was scored in a blinded manner based on the percentage of tubules showing dilation, flattening, cast formation, or necrosis in 10 randomly selected non-overlapping cortical fields per kidney under 200× magnification. The injury grading was defined as follows: 0 (none), 1 (≤10%), 2 (11–25%), 3 (26–50%), 4 (>50%) [32].

### 2.5 Immunohistochemistry and immunofluorescence

Paraffin sections were deparaffinized, rehydrated and subjected to antigen retrieval. After blocking, sections were incubated with primary antibodies overnight at 4°C. For immunohistochemistry, HRP-conjugated secondary antibodies were applied, followed by DAB development. For immunofluorescence, section was incubated with Alexa Fluor 594-conjugated secondary antibodies. Renal tubules were specifically labeled with *Lotus tetragonolobus* lectin (LTL, Vector Laboratories). Images were acquired with a bright-field or fluorescence microscope, and ten random fields per section were selected for quantification. Primary antibody information is detailed in Supplementary Table 6.

### 2.6 Cell viability assay

Cell viability was measured by CCK-8 kit. Cells were seeded in 96-well plates and treated according to experimental design. To examine the effect of ferroptosis, cells overexpressing PRMT5 were co-treated with 10 μM ferrostatin-1 (Fer-1), 20 μM Z-VAD-FMK (apoptosis inhibitor), 10 μM necrostain-1 (necroptosis inhibitor, Nec-1), or 5 mM 3-MA (autophagy inhibitor). In parallel, PRMT5-knockdown cells were treated with 0.1 μM RSL3 or 10 μM erastin. After treatment, 10 μL CCK-8 reagent was added per well and incubated for 2 h at 37°C. Absorbance was measured at 450 nm using a microplate reader.

### 2.7 Western blot analysis

Proteins were extracted from cells or kidney tissues using RIPA lysis buffer containing protease inhibitors. BCA Protein Assay Kit was then used to measure protein concentration. Equal amounts of protein were separated by SDS-PAGE and transferred onto NC membranes. Membranes were blocked with 5% non-fat milk for 1 h and incubated overnight at 4°C with primary antibodies. After washing, membranes were incubated with HRP-conjugated secondary antibodies for 1 h at room temperature. Protein bands were visualized using an ECL detection system. Primary antibody information is detailed in Supplementary Table 6.

### 2.8 RNA extraction and RT-qPCR

Total RNA was extracted from cells using a column-based kit. cDNA was synthesized from 1 μg RNA with a reverse transcription kit. Quantitative PCR was performed in triplicate using SYBR Green master mix on a real-time PCR system. The 2^−ΔΔCt^ method was used for relative quantification, with GAPDH as the internal control. Primer sequences are listed in Supplementary Table 7.

### 2.9 TUNEL assay

Cell death was assessed by TUNEL staining: One-Step TUNEL Apoptosis Assay Kit for cultured cells and DAB TUNEL Detection Kit for paraffin-embedded kidney tissues. All samples were fixed, permeabilized, and incubated with the respective TUNEL reaction mixture for 1 h at 37°C in the dark, with tissue sections additionally deparaffinized and proteinase K-treated prior to incubation. Images were acquired using a fluorescence microscope and analyzed with ImageJ software to quantify the percentage of TUNEL-positive cells.

### 2.10 EdU proliferation assay

Cell proliferation was evaluated using an EdU assay kit. Cells were seeded in 24-well plates and incubated with 10 μM EdU for 2 h at 37°C. After fixation and permeabilization, cells were reacted with the Click reaction cocktail for 30 min in the dark. Nuclei were counterstained with Hoechst 33342. Images were acquired using a fluorescence microscope, and EdU-positive cells were quantified with ImageJ software.

### 2.11 Measurement of MDA, GSH levels and GSH/GSSG ratio

Malondialdehyde (MDA) content in kidney tissues and HK-2 cells were determined using a Lipid Peroxidation MDA Assay Kit according to the manufacturer's instructions. Total glutathione (GSH) and the reduced-to-oxidized glutathione ratio (GSH/GSSG) were measured with a Glutathione Assay Kit. Homogenates were prepared in the provided assay buffer, and absorbance was recorded at 532 nm for MDA and 412 nm for GSH/GSSG using a microplate reader. All values were normalized to total protein concentration measured by a BCA protein assay kit.

### 2.12 Lipid peroxidation assay

Lipid peroxidation was assessed with C11-BODIPY probe. Samples were loaded with 5 μM C11-BODIPY for 30 min at 37°C. For imaging, red (590 nm, non-oxidized) and green (510 nm, oxidized) fluorescence was captured under microscope and quantified with ImageJ. For quantitative profiling, cells were analyzed by flow cytometry in the FITC channel. Mean fluorescence intensity (MFI) was evaluated by FlowJo software.

### 2.13 Intracellular labile iron

Intracellular labile iron levels were assessed using FerroOrange probes. HK-2 cells were incubated with 1 μM FerroOrange for 30 min at 37°C in the dark and immediately analyzed. All samples were detected using a flow cytometer. MFI was evaluated by FlowJo software.

### 2.14 Mito-FerroGreen measurement

Mitochondrial labile iron levels were assessed using Mito-FerroGreen probes. HK-2 cells were incubated with 5 μM Mito-FerroGreen for 30 min at 37°C in the dark. Without washing, the cells were immediately analyzed using a flow cytometer. MFI in the FITC channel was evaluated using FlowJo software to determine mitochondrial ferrous iron content.

### 2.15 Mitochondrial ROS detection

Mitochondrial superoxide was detected using MitoSOX Red reagent. Cells were incubated with 5 μM MitoSOX for 30 min at 37°C, washed with PBS, and immediately imaged under a fluorescence microscope. Fluorescence intensity was quantified with ImageJ software.

### 2.16 ATP measurement

Cellular ATP levels were measured using an ATP Assay Kit. Cells were lysed according to the manufacturer's protocol, and luminescence was recorded with a microplate reader. ATP concentrations were normalized to total protein content.

### 2.17 Transmission electron microscopy

Cells were prefixed in cold 3% glutaraldehyde for 2 h at 4°C, followed by post-fixation in 1% osmium tetroxide. Samples were dehydrated through a graded ethanol series and embedded in epoxy resin. Ultrathin sections were stained with uranyl acetate and lead citrate. Mitochondrial ultrastructure was observed and imaged using a transmission electron microscope. For morphometric analysis, individual mitochondrial cross-sectional areas were calculated using ImageJ. Mitochondrial cristae injury was semi-quantitatively scored on a 0 – 3 scale in a blinded manner by two independent investigators: 0 (intact), 1 (mild damage, <30% loss), 2 (moderate damage, 30%–70% loss), and 3 (severe damage, >70% loss or rupture). Three independent samples per group were evaluated, with 10 individual mitochondria analyzed per sample [33].

### 2.18 RNA sequencing

Total RNA was extracted from HK-2 cells treated with cisplatin or cisplatin combined with PRMT5 knockdown using TRIzol reagent. RNA samples were then submitted to LC-Biotech Corporation for library preparation and sequencing on an Illumina NovaSeq 6000 platform. Differentially expressed genes (DEGs) between groups were identified using the DESeq2 R package, with screening thresholds set at *P* < 0.05 and │log_2_ Fold Change│> 1. Gene Ontology (GO) functional enrichment analysis was performed on DEGs using the clusterProfiler R package, and term with *P* < 0.05 were considered significantly enriched. Gene Set Enrichment Analysis (GSEA) was performed on the normalized expression data using GSEA software (v4.3.2) and the MSigDB hallmark gene sets (v2023.1). Gene sets with │NES│ > 1 and FDR < 0.05 were considered statistically significance.

### 2.19 CUT&TAG

CUT&TAG libraries were constructed using CUT&TAG Library Kit according to the manufacturer's protocol. Briefly, HK-2 cells were bound to concanavalin A beads, permeabilized with digitonin, and incubated with H3K4me3 primary antibodies and corresponding secondary antibody. Cell were then incubated with pA-Tn5 transposase for targeted tagmentation. The fragments were extracted and amplified by PCR. Libraries were sequenced on an Illumina NovaSeq 6000 platform. For data analysis, raw reads were aligned to the GRCh38 genome using Bowtie2. H3K4me3 binding peaks were identified using SEACR software under the tringent parameter. Peaks annotation and global distributions were calculated using the ChIPseeker R package. H3K4me3 enrichment on the *ACSL4* locus was visualized using the Integrative Genomics Viewer (IGV).

### 2.20 CUT&RUN-qPCR

CUT&RUN was performed using a kit following the manufacturer's protocol. HK-2 cells were bound to concanavalin A beads, permeabilized, and incubated with anti-H3K4me3 or anti-H3R2me2s or anti-WDR5 or control IgG antibodies. Cells were then treated with pAG-MNase for targeted chromatin digestion. After DNA extraction and purification, qPCR was conducted using specific primer pairs targeting the *ACSL4* promoter regions. Data were normalized to input DNA and expressed as fold enrichment. All CUT&RUN-qPCR assays were obtained from n = 3 independent biological experiments, with technical triplicates averaged for each biological sample for statistical analysis. The primer sequences are listed in Supplementary Table 8.

### 2.21 Statistical analysis

Statistical analyses were performed using GraphPad Prism 9. Data normality was assessed using Shapiro-Wilk, and homogeneity of variance was evaluated using Brown-Forsythe test. For comparisons between two groups with normal distribution, Student's *t*-test was applied. Comparisons among more than two groups with normal distribution and equal variance were conducted using one-way ANOVA followed by Tukey's post-hoc test. For non-normally distributed data, the Mann-Whitney test was used for two groups comparison, the Kruskal-Wallis test followed by Dunn's post-hoc test was used for multiple group comparisons. Quantitative data are presented as mean ± SD. P-value < 0.05 was considered statistically significant.

## 3 Results

### 3.1 PRMT5-MEP50 is upregulated in tubular epithelial cells across diverse AKI models

To explore the potential role of PRMT5 in AKI, we performed immunohistochemistry (IHC) on human kidney biopsy specimens. PRMT5 expression was minimal in healthy para-renal control tissues, but increased in TECs of AKI patients (Fig. 1A). In CP-AKI model, a single dose of cisplatin resulted in acute renal failure, with SCr and BUN rising 2 to 3-fold within 72 h (Fig. 1B). H&E and PAS staining revealed prominent tubular injury, including tubular dilation, cast formation, loss of the brush border, and epithelial shedding, leading to a significant increase in tubular injury scores in CP group (Fig. 1C). IHC and Western blot analyses showed a dose-dependent increased of PRMT5 and its cofactor MEP50 in the kidney cortex. Pearson's correlation analysis showed that PRMT5/MEP50 protein levels correlated positively with SCr, suggesting that PRMT5 might reflect tubular damage (Fig. 1D). Accompanying this upregulation, Immunofluorescence (IF) co-staining with NGAL confirmed that PRMT5 upregulation was mainly in damaged proximal tubules (Fig. 1E). Western blot analysis showed that the expression of symmetric dimethylarginine (SDMA), the catalytic product of PRMT5, was also elevated in the injured kidneys (Fig. 1F). A similar activation patterns were observed in S-AKI and FA-AKI mice models. In these models, the induction of renal dysfunction and pathological damage was closely mirrored by an increase in renal PRMT5 expression, as confirmed by biochemical and histopathological assessments (Supplementary Fig. 1A–H). To assess whether PRMT5 showed prominent response among PRMT family members, PRMT family expression was analyzed in public datasets from mouse kidney tissue under cisplatin, ischemia-reperfusion (IRI), and LPS induced injury models. Among all family members, *Prmt5* was identified as a commonly and significantly upregulated gene across different stress conditions (Supplementary Fig. 2A). To validate this findings in vitro, RT-qPCR was performed in HK-2 cells under cisplatin, hypoxia/reoxygenation (H/R), and LPS stimulation. The results confirmed that *PRMT5* exhibited the most consistent and significant upregulation across all conditions (Supplementary Fig. 2B). Western blot analysis demonstrated a dose-dependent increase in PRMT5, MEP50, and SDMA protein levels following cisplatin stimulation in HK-2 cells (Fig. 1G), which was also consistently observed in cisplatin-treated NRK cells (Supplementary Fig. 2I). Furthermore, IF staining showed an enhanced nuclear accumulation of both PRMT5 and SDMA in cisplatin treated HK-2 cells (Supplementary Fig. 3A and B). These findings indicate that PRMT5 is a nuclear responder, which is selectively upregulated in TECs following diverse renal insults.

**Fig. 1:**
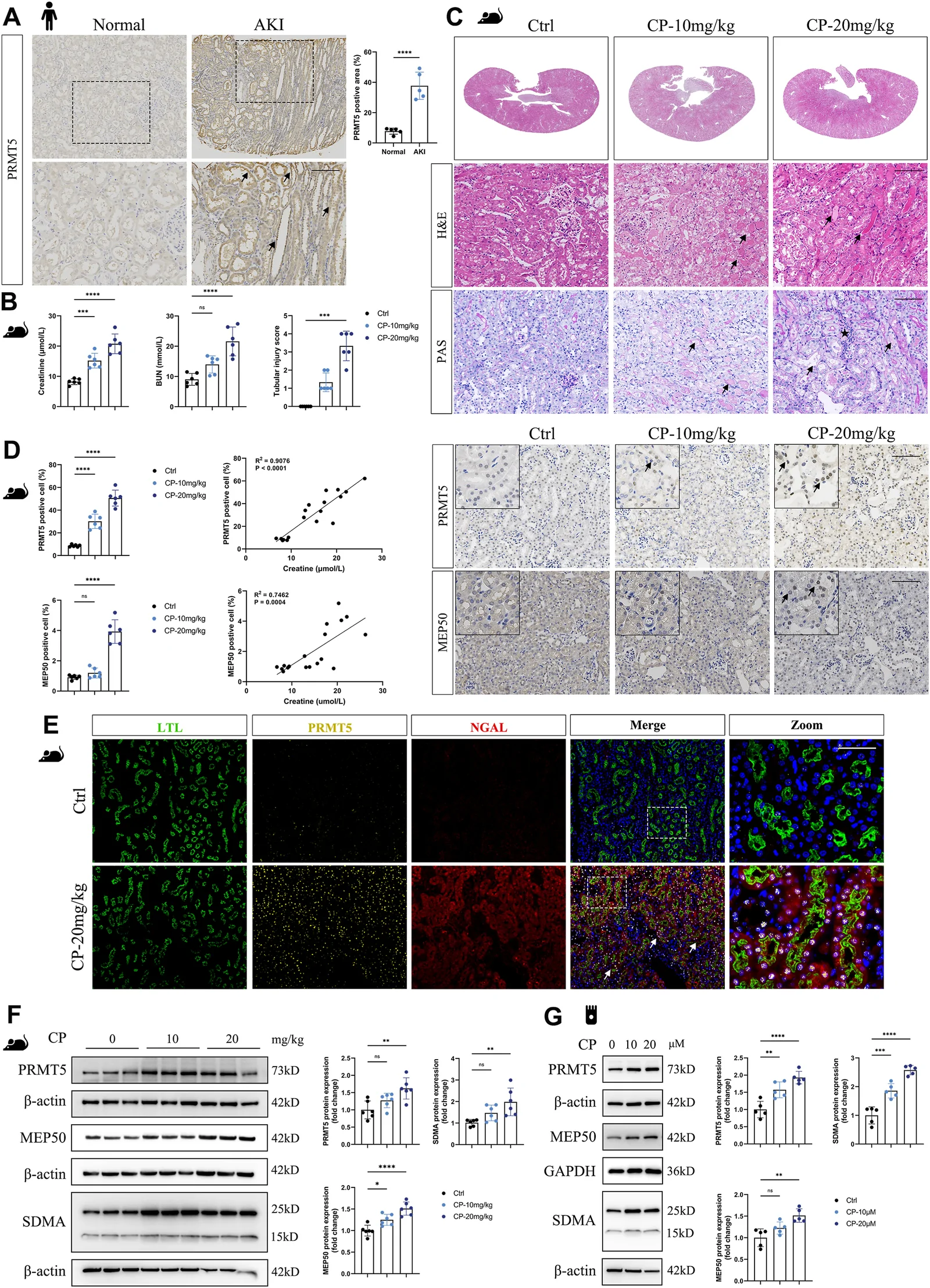
PRMT5 expression is elevated in tubular epithelial cells of AKI. A. Representative immunohistochemical (IHC) staining of PRMT5 in kidney biopsy specimens from patients with AKI, and para-carcinoma renal tissue as control group. Black arrows indicate PRMT5 positive epithelial cells, n = 5 in each group. (B-F) C57BL/6 mice were intraperitoneal (i.p.) injection of 10 or 20 mg/kg cisplatin for 72 h, n = 6 biological replicates in each group.B. SCr and BUN levels in different groups of cisplatin-induced AKI (CP-AKI) mice.C. Representative images of H&E and PAS staining showing tubular injury in kidneys, and histopathology score of tubular damage. Black arrows indicate tubular dilation, cast formation, loss of brush border and epithelial shedding. Black asterisks indicate inflammatory cell infiltration. Scale bars, 100 μm.D. IHC analysis and quantification of PRMT5 and its co-factor MEP50. Black arrows indicate the nuclei of epithelial cells stained for PRMT5 (upper) or MEP50 (lower). Pearson's correlation between PRMT5/MEP50 levels and SCr. Scale bars, 50 μm E. Representative images of PRMT5 (yellow), LTL (green), NGAL (red) and DAPI (blue) immunofluorescence in CP-AKI mice kidney tissue. White arrows indicate the co-localization of PRMT5 within NGAL positive epithelial cells in damaged tubules. Scale bars, 3 μm F. Western blot analysis and quantification of PRMT5, co-factor MEP50 and SDMA in kidney tissue of CP-AKI mice.G. Western blot analysis and quantification of PRMT5, MEP50 and SDMA in HK-2 cells treated with indicated concentrations of cisplatin, n = 5 independent biological experiments. Data are presented as mean ± SD. Significance was assessed by one-way ANOVA with Tukey's post-hoc test or unpaired Student's *t*-test. The tubular injury score was analyzed using the non-parametric Kruskal-Wallis test followed by Dunn's post-hoc test. NS, indicates no significant, *P < 0.05, **P < 0.01, ***P < 0.005, ****P < 0.001.

### 3.2 PRMT5 depletion attenuates tubular cell injury in vitro and preserves renal function in CP-AKI mice

To determine whether the observed upregulation of PRMT5 is a functional driver of tubular injury, gain and loss of function assays were performed in HK-2 cells.

First, PRMT5 was silenced using specific siRNA, which caused a significant reduction in both mRNA (Fig. 2A) and protein levels (Fig. 2B). The selected siRNA-mediated knockdown of PRMT5 significantly enhanced cell viability following cisplatin challenge (Fig. 2C). Western blot analysis further revealed that PRMT5 deficiency attenuated the expression of the injury marker NGAL, accompanied by a decrease in SDMA modified protein levels (Fig. 2D). TUNEL staining revealed a significantly lower percentage of cell death in siPRMT5 group compared to the scramble group (Fig. 2E). Additionally, EdU assays demonstrated that PRMT5 deficiency increased the compensatory proliferative capacity of TECs following injury (Fig. 2F). Conversely, the impact of PRMT5 overexpression (OE) was evaluated using a stable lentiviral-mediated HK-2 cell line. The overexpression was confirmed by an increase in mRNA (Fig. 2G) and protein expression (Fig. 2H). Under cisplatin challenge, overexpressing PRMT5 resulted in significantly impaired cell viability (Fig. 2I). Furthermore, PRMT5 overexpression resulted in increased TUNEL positive cell death (Fig. 2J) and severely suppressed cellular proliferation as evidenced by EdU staining (Fig. 2K).

**Fig. 2:**
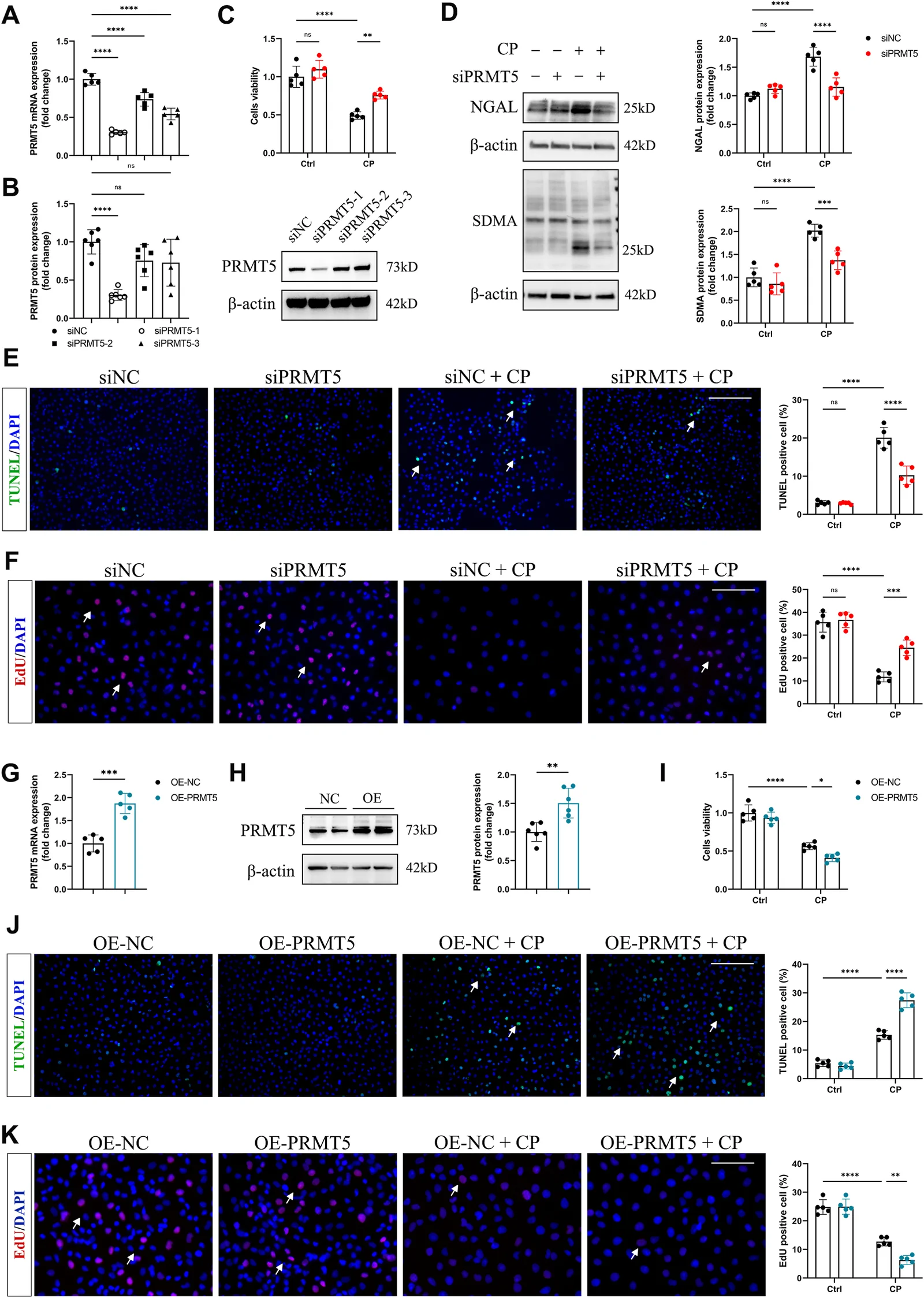
Modulation of PRMT5 expression alters injury sensitivity in vitro. (A-F) HK-2 cells were transfected with PRMT5 siRNA followed by 20 μM cisplatin treatment for 24 h, n = 5-6 independent biological experiments. A. Relative PRMT5 mRNA expression.B. Western blot analysis and quantification of PRMT5 expression in cells.C. Assessment of cell viability after PRMT5 knockdown.D. Western blot analysis and quantification of NGAL and SDMA expression.E. Representative images of TUNEL staining and apoptosis cells quantification. White arrows indicate TUNEL positive nuclei. Scale bar, 250 μm F. Representative images of EdU staining and cell proliferation analysis. White arrows indicate EdU positive nuclei in cells following cisplatin exposure. Scale bar, 100 μm.(G-K) HK-2 cells were transduced with PRMT5 overexpressing lentivirus, followed by cisplatin treatment for 24 h, n = 5-6 independent biological experiments.G. Relative PRMT5 mRNA expression in PRMT5 overexpressing cells.H. Western blot analysis and quantification of PRMT5 expression in cells.I. Assessment of cell viability after PRMT5 over-expression.J. Representative TUNEL staining images and apoptosis cells quantification. White arrows indicate TUNEL positive nuclei. Scale bar, 250 μm K. Representative EdU staining images and cell proliferation analysis. White arrows indicate EdU positive nuclei in cells following cisplatin exposure. Scale bar, 100 μm. Data are presented as mean ± SD. Technical replicates were averaged for analysis. Significance was assessed by one-way ANOVA with Tukey's post-hoc test or unpaired Student's *t*-test. NS, indicates no significant, *P < 0.05, **P < 0.01, ***P < 0.005, ****P < 0.001.

To extend in vitro findings to a systemic context, a renal tubule targeted knockdown was achieved in mice via retrograde renal pelvic injection of an AAV-shPRMT5 vector (Fig. 3A). To confirm the technical success and efficiency of this injection, Western blot analysis verified that AAV-shPRMT5 administration led to a substantial reduction in PRMT5 protein levels (Fig. 3B). Under cisplatin stimulation, mice receiving AAV-shPRMT5 maintained a remarkable preservation on renal function, as evidenced by lower SCr and BUN levels (Fig. 3C). Kidney weight loss was also prevented in PRMT5 depletion mice (Fig. 3D). Histological analysis showed markedly preserved tubular architecture with reduced cell degeneration, tubular dilation, and cast formation, resulting in decreased tubular injury scores in AAV-shPRMT5 mice. Beyond structural preservation, the percentage of TUNEL positive cell was reduced in the knockdown group (Fig. 3E). PRMT5 knockdown further attenuated the inflammatory response triggered by cisplatin, shown by reduced interstitial infiltration of F4/80+ macrophages and Ly6g + neutrophils (Fig. 3F). Western blot analysis of the kidney cortex confirmed that the genetic loss of PRMT5 led to a substantial reduction in the expression of NGAL and KIM-1, along with reduced levels of SDMA modified proteins (Fig. 3G). Together, these results demonstrate that tubule specific PRMT5 depletion confers structural and functional protection against CP-AKI, underscoring its role as a key mediator of renal injury.

**Fig. 3:**
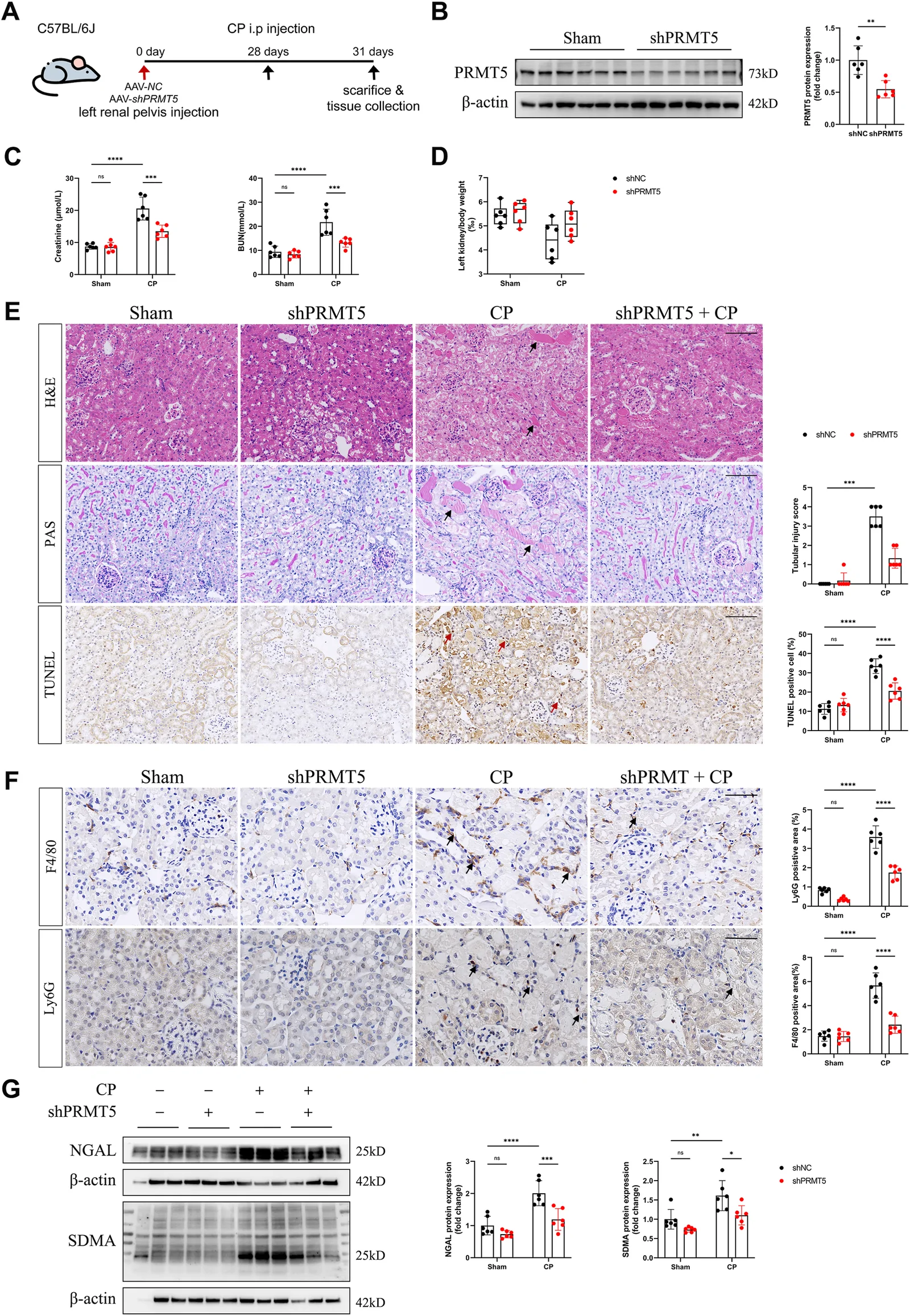
Genetic depletion of PRMT5 preserves renal function and ameliorated tubular injury in CP-AKI mice. A. Schematic of the experimental design for AAV-mediated PRMT5 knockdown. AAV-shPRMT5 or control vector (AAV-NC) was administered by renal pelvic injection to achieve targeted PRMT5 knockdown in the renal tubule. After 4 weeks, mice received a single dose of cisplatin. B. Western blot analysis and quantification of PRMT5 expression in kidney cortical lysates. C. SCr and BUN levels. D. Fig. 3D. Left kidney to body weight ratio after cisplatin treatment (5.100 ± 0.607‰ in the shPRMT5 + CP group vs. 4.385 ± 0.784‰ in the CP group). E. Representative H&E, PAS and TUNEL staining images. Right panels show tubular damage score and quantification. Black arrows highlight tubular dilation, cast formation, loss of brush border and epithelial shedding, red arrows indicate death of tubular epithelial cells. Scale bars, 100 μm. F. Representative F4/80 and Ly6g staining images and quantification analysis. Black arrows indicate F4/80 positive macrophages or Ly6G positive neutrophils infiltrating the tubulointerstitial are. Scale bars, 50 μm. G. Western blot analysis and quantification of tubular injury markers NGAL and SDMA modification expression in kidney cortical lysates. Data are presented as mean ± SD, n = 6 biological replicates in each group. Significance was assessed by one-way ANOVA with Tukey's post-hoc test or unpaired Student's *t*-test. The tubular injury score was analyzed using the non-parametric Kruskal-Wallis test followed by Dunn's post-hoc test. NS, indicates no significant, *P < 0.05, **P < 0.01, ***P < 0.005, ****P < 0.001.

### 3.3 PRMT5 knockdown inhibited lipid peroxidation and ferroptotic death both in cisplatin induced HK-2 cells and CP-AKI mice

To define the cell death pathway regulated by PRMT5, pharmacological inhibitors targeting distinct death modalities were applied in cisplatin treated HK-2 cells. In PRMT5-OE group, inhibition of apoptosis (Z-VAD-FMK), necroptosis (Nec-1), autophagy (3-MA) and ferroptosis (Fer-1) each partially restored cell viability. Among these, both the ferroptosis inhibitor Fer-1 and the necroptosis inhibitor Nec-1 exhibited pronounced protective effects, with Fer-1 conferring the most substantial rescue of cell viability compared to the other compounds (Fig. 4A). Consistently, PRMT5 knockdown markedly increased cellular resistance to the ferroptosis inducer RSL3 or erastin, further confirming the specific involvement of ferroptosis (Fig. 4B). Biochemical features characteristic of ferroptosis were subsequently evaluated. PRMT5 knockdown significantly attenuated lipid peroxidation, as indicated by reduced oxidation of C11-BODIPY immunofluorescence staining (Fig. 4E), and confirmed by flow cytometric quantification of oxidized C11-BODIPY (Fig. 4F). Consistently, the MDA content accumulation was markedly decreased upon PRMT5 deficiency (Fig. 4C). PRMT5 deficiency enhanced resistance to oxidative stress, as reflected by increased total GSH levels and a significantly improved GSH/GSSG ratio (Fig. 4D). To explore the underlying molecular changes, the transcriptional profiles of ferroptosis-related genes were screened firstly. It revealed that PRMT5 knockdown significantly regulated their mRNA levels (Supplementary Fig. 4). Consistent with these transcriptional shifts, Western blot analysis showed that PRMT5 knockdown increased the expression of antioxidant proteins, including NRF2, GPX4, and xCT. Meanwhile, the major ferroptosis driver ACSL4 expression was significantly suppressed (Fig. 4G). In short, these results showed PRMT5 knockdown prevented lipid peroxidation and stabilized the antioxidant system in cisplatin-induced HK-2 cells.

**Fig. 4:**
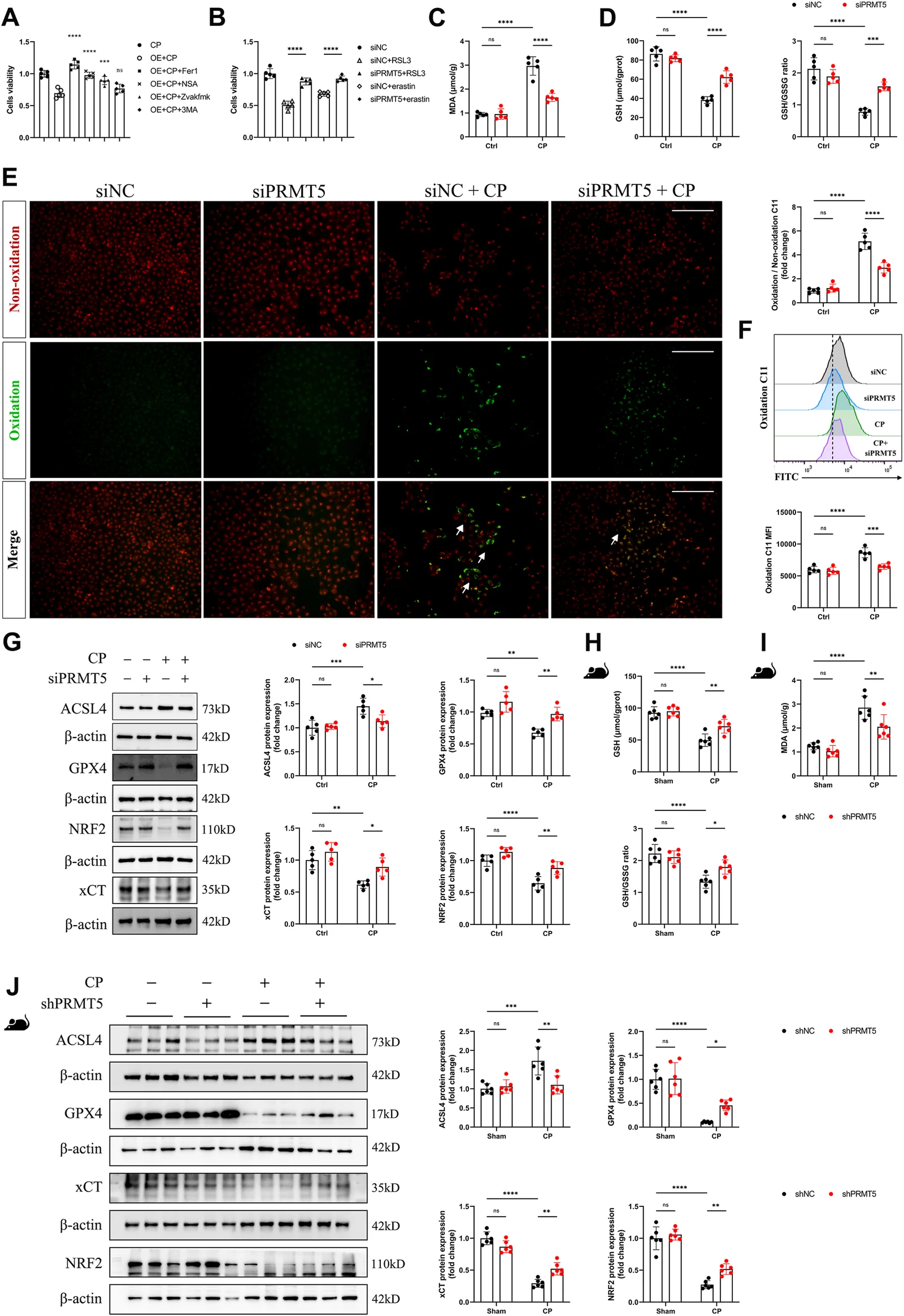
PRMT5 knockdown inhibited lipid peroxidation and ferroptotic death both in cisplatin-induced HK-2 cells and CP-AKI mice A. Cell viability assessment. PRMT5 overexpressing HK-2 cells were treated with various cell death inhibitors, followed by 20 μM cisplatin treatment for 24 h, n = 5 independent biological experiments. B. Cell viability assessment. PRMT5 knockdown HK-2 cells were treated with ferroptosis inducers (0.1 μM RSL3 or 10 μM erastin) for 24 h, n = 5 independent biological experiments. (C- I) HK-2 cells were exposed to 20 μM cisplatin for 24 h after siRNA transfection, n = 3-5 independent biological experiments.C. MDA content.D. GSH levels and GSH/GSSG ratio in HK-2 cells.E. Representative images and quantification of C11-BODIPY staining. Non-oxidized stained in red signals and oxidized stained in green signals. Scale bars, 250 μm F. Flow cytometric analysis of lipid peroxidation using C11-BODIPY.G. Western blot analysis and quantification of ACSL4, GPX4, NRF2 and xCT protein expression.(H-J) Mice received AAV-NC or AAV-shPRMT5 injection were treated with cisplatin for 72 h, n = 6 biological replicates in each group.H. GSH levels and GSH/GSSG ratio in the renal cortex.I. MDA contents in the renal cortex.J. Western blot analysis and quantification of the expression ACSL4, GPX4, NRF2 and xCT protein expression. Data are presented as mean ± SD. Technical replicates were averaged for analysis, and significance was assessed by one-way ANOVA with Tukey's post-hoc test. NS, indicates no significant, *P < 0.05, **P < 0.01, ***P < 0.005, ****P < 0.001.

To validate the anti-ferroptotic effect of PRMT5 knockdown in vivo, renal cortical tissue were analyzed from CP-AKI mice. IHC staining revealed robust accumulation of 4-hydroxynonenal (4HNE) and ACSL4 in AKI mice. In contrast, AAV-shPRMT5 mice showed a significant reduction in the levels of both markers (Supplementary Fig. 5A). The reduction in 4-HNE accumulation was also confirmed by immunofluorescence staining (Supplementary Fig. 5B). Consistent with these histological changes, the MDA content in the renal cortex was lowered and the GSH/GSSG ratio was significantly improved in PRMT5-knockdown mice compared to the counterparts (Fig. 4H and I). Correspondingly, Western blotting demonstrated that PRMT5 depletion not only suppressed the pro-ferroptotic regulator ACSL4, but also rescued the NRF2/GPX4/xCT antioxidant defense axis in kidney tissue (Fig. 4J). These results demonstrated that PRMT5 drove AKI by tipping the balance of lipid peroxidation, antioxidant defense toward a pro-ferroptotic state.

### 3.4 PRMT5 depletion restores iron homeostasis and mitigates mitochondrial damage in tubular cells

We next examined whether PRMT5 depletion affected iron homeostasis and mitochondrial function during cisplatin induced injury. Flow cytometric analysis using FerroOrange revealed that cisplatin challenge triggered a prominent accumulation of intracellular labile iron, which was significantly rescued by PRMT5 silencing (Fig. 5A). Consistently, Mito-FerroGreen staining further revealed that PRMT5 silencing also reduced the cisplatin-induced accumulation of mitochondrial labile iron (Fig. 5B). Meanwhile, the iron-storage protein FTH expression was elevated, whereas the levels of the iron-uptake protein TFRC were significantly suppressed under PRMT5 knockdown condition both in vitro and in vivo (Fig. 5C and D). Theses findings suggested that PRMT5 depletion may help maintain cellular iron balance under cisplatin stress. Furthermore, the effects of PRMT5 on mitochondrial function were analyzed. The loss of intracellular ATP production was successfully restored in PRMT5-deficient cells (Fig. 5E). Consequently, cisplatin challenge significantly increased mitochondrial superoxide production, which was largely suppressed by PRMT5 knockdown (Fig. 5F). Moreover, TEM revealed that PRMT5 knockdown preserve mitochondrial ultrastructure, preventing mitochondrial shrinkage, increase membrane density, and cristae loss induced by cisplatin. Quantitative analysis further showed that PRMT5 deficiency preserved the average mitochondrial area and significantly decreased mitochondria cristae integrity score (Fig. 5G).

**Fig. 5:**
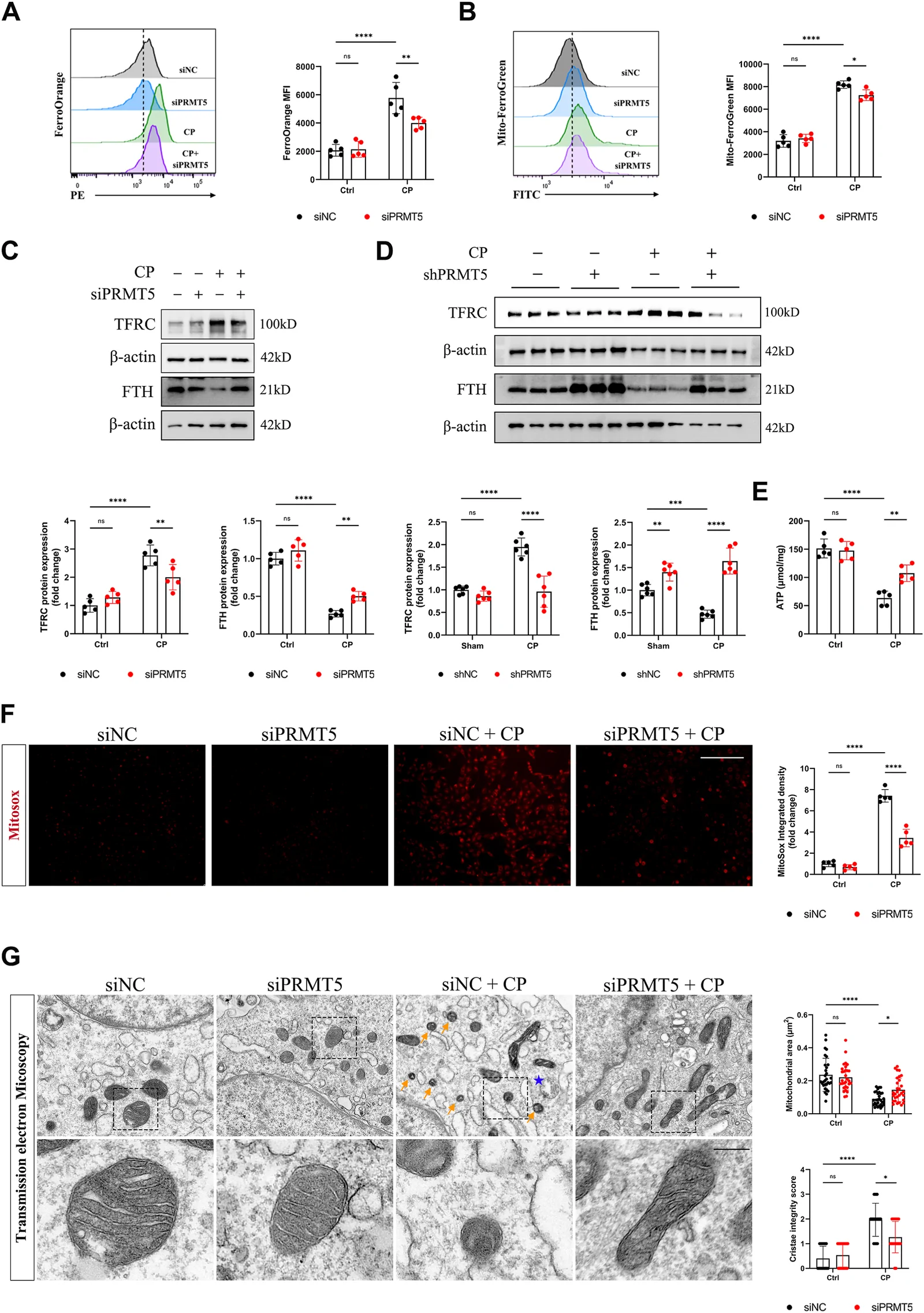
PRMT5 knockdown ameliorated iron metabolism dyshomeostasis and restored mitochondrial function in cisplatin-treated HK-2 cells.HK-2 cells were exposed to 20 μM cisplatin for 24 h after siRNA transfection, n = 5 independent biological experiments. A. Flow cytometric analysis of labile iron pool using FerroOrange.B. Flow cytometric analysis of Mito-FerroGreen fluorescence in HK-2 cells.C. Western blot analysis and quantification of TFRC and FTH protein expression.D. Western blot analysis and quantification of TFRC and FTH protein expression in CP-AKI mice, n = 6 biological replicates in each group.E. ATP production.F. Representative images and quantification of MitoSOX staining. Scale bars, 250 μm G. Representative transmission electron microscopy (TEM) images. Yellow arrows indicate shrunken mitochondria with increased membrane density and loss of cristae; blue stars indicate the lysed mitochondria. For morphometric analysis, mitochondrial area and mitochondrial cristae integrity score were calculated, n = 3 independent samples per group, with 10 individual mitochondria analyzed per sample. Scale bars, 250 nm. Data are presented as mean ± SD. Significance was assessed by one-way ANOVA with Tukey's post-hoc test. The mitochondrial cristae integrity score was analyzed using the non-parametric Kruskal-Wallis test followed by Dunn's post-hoc test. NS indicates no significant, *P < 0.05, **P < 0.01, ***P < 0.005, ****P < 0.001. NS indicates not significant.

### 3.5 PRMT5 regulates Acsl4 epigenetic activation through WDR5 mediated H3K4me3 enrichment

To investigate how PRMT5 regulated ferroptosis in AKI, a differential gene expression analysis was performed in HK-2 cells treated with cisplatin, comparing PRMT5 knockdown. The Gene Ontology (GO) analysis revealed a significant enrichment of terms related to transcriptional regulation, supporting a role for PRMT5 in governing gene expression at transcriptional level during cisplatin stimulation (Supplementary Fig. 6). To further identify the specific epigenetic programs modulated by PRMT5 depletion under cisplatin stress, Gene Set Enrichment Analysis (GSEA) was performed and highlighted that H3K4 trimethylation (H3K4me3) related gene signatures were significantly altered downstream upon PRMT5 knockdown (Fig. 6A). Because H3K4me3 is a canonical active transcription mark catalyzed by the MLL/WDR5 complex, and PRMT5 mediated arginine methylation has been reported to cross talk with H3K4me3 machinery [34]. Based on these bioinfrmatic clues, we hypothesized that PRMT5 influenced chromatin accessibility and H3K4me3 deposition through WDR5 associated complex during renal injury (Fig. 6B). Western blot analysis showed that cisplatin-induced upregulation of PRMT5 was accompanied by an increase in histone modification, especially H3K4me3 and H3R2me2s modifications in mice kidney tissues (Fig. 6C) and HK-2 cells (Supplementary Fig. 7). Crucially, PRMT5 knockdown led to a marked reduction in these histone modifications (Fig. 6D). To further map the global distribution of H3K4me3, we performed CUT&TAG sequencing. The heatmap and pie charts illustrated distinct alterations in global H3K4me3 binding peaks and genomic distribution upon cisplatin treatment (Fig. 6e and F). Specifically, IGV tracks revealed a prominent enrichment of H3K4me3 on the *ACSL4* promoter region (Fig. 6G). Finally, at the local promoter level, CUT&RUN-qPCR analysis confirmed that cisplatin stimulation significantly induced H3K4me3, H3R2me2s, and WDR5 occupancy on the *ACSL4* promoter, whereas PRMT5 knockdown markedly reduced their enrichment (Fig. 6H). These findings indicate that PRMT5 knockdown is accompanied by a concurrent loss of local dimethylation marks, reduced WDR5 binding, and decreased H3K4me3 enrichment at the *ACSL4* promoter, supporting a functional association between PRMT5 status and local chromatin accessibility.

**Fig. 6:**
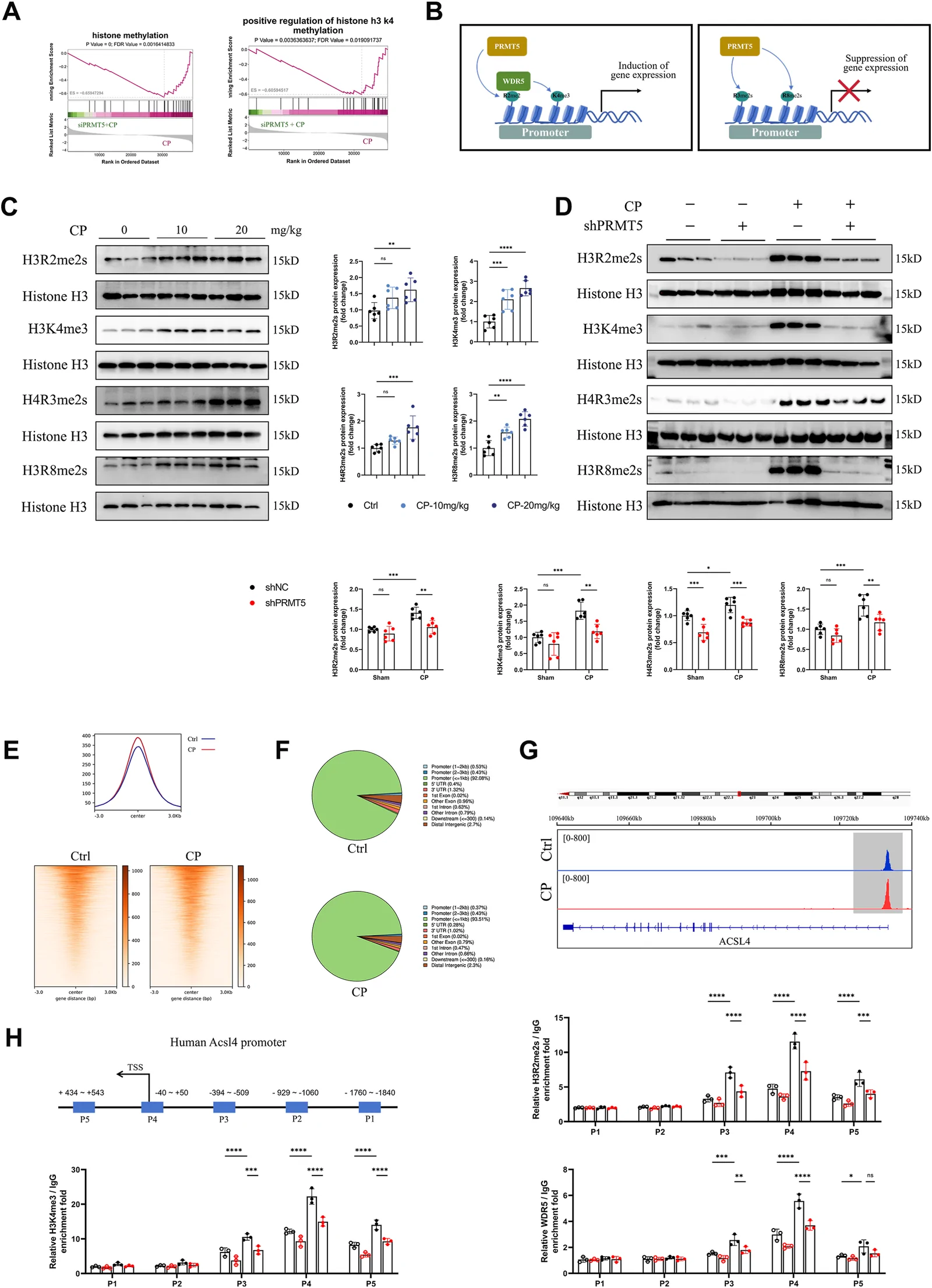
PRMT5 influence H3K4me3 enrichment on *ACSL4* promoter. A. GSEA enrichment plot showing the correlation between PRMT5 and histone modification related pathway. B. Schematic diagram illustrating the molecular mechanism of PRMT5-mediated histone modification. C. Western blot analysis of histone modifications from CP-AKI mice kidney tissue, n = 6 individual mouse samples per group. D. Western blot analysis of histone modifications in PRMT5 knockdown mice, n = 6 individual mouse samples per group. E. Heatmap illustrating the global distribution of H3K4me3 binding peaks with or without 20 μM cisplatin treatment in HK-2 cells, n = 2 independent biological experiments in each group. F. Pie charts showing the genomic distribution of H3K4me3 peaks in Ctrl and CP treated cells. G. IGV tracks showing H3K4me3 enrichment on *ACSL4* promoter in HK-2 cells. The *ACSL4* gene is located on the minus strand. Arrows indicate the transcription direction and promoter region of *ACSL4*. H. CUT&RUN-qPCR validation of H3K4me3, H3R2me2s and WDR5 enrichment on the *ACSL4* promoter in HK-2 cells, n = 3 independent biological experiments, with technical triplicates averaged for each biological sample. The *ACSL4* gene is located on the minus strand. Arrows indicate the transcription direction and promoter region of *ACSL4*. Data are presented as mean ± SD. Significance was assessed by one-way ANOVA with Tukey's post-hoc test or unpaired Student's *t*-test. NS, indicates no significant, *P < 0.05, **P < 0.01, ***P < 0.005, ****P < 0.001.

Having established the presence of WDR5 at the *ACSL4* promoter, we next evaluated whether WDR5 expression is upregulated during AKI and functionally required for ferroptotic injury. At the protein expression, IHC analysis showed that both WDR5 and MLL1 were highly expressed in the injured tubules of CP-AKI mice (Fig. 7A). Western blot analysis confirmed that cisplatin challenge significantly increased the protein levels of WDR5 and MLL1 both in vitro (Fig. 7B) and in vivo (Fig. 7C). To investigate the functional role of WDR5 in tubular death, HK-2 cells were transfected with WDR5 siRNA prior to cisplatin treatment. Transfection efficiency was verified by a significant decrease in *WDR5* mRNA (Fig. 7D) and protein levels (Fig. 7E). In functional assays, WDR5 knockdown successfully restored cell viability under cisplatin condition (Fig. 7F). In addition, WDR5 knockdown reduced MDA content (Fig. 7G), and increased intracellular GSH levels (Fig. 7H) in cisplatin-treated cells. Furthermore, silencing WDR5 mimicked the effects of PRMT5 knockdown by downregulating both H3K4me3 and the pro-ferroptotic driver ACSL4 protein expression (Fig. 7I). To assess the functional cooperation between PRMT5 and WDR5 on *ACSL4* transcription, co-manipulation experiments were performed. While PRMT5 overexpression strongly increased *ACSL4* mRNA levels, this effect was significantly blocked by WDR5 knockdown (Fig. 7J). Together, these results supported a cooperative role for PRMT5 and WDR5 in driving ACSL4 expression and tubular cell ferroptosis during CP-AKI.

**Fig. 7:**
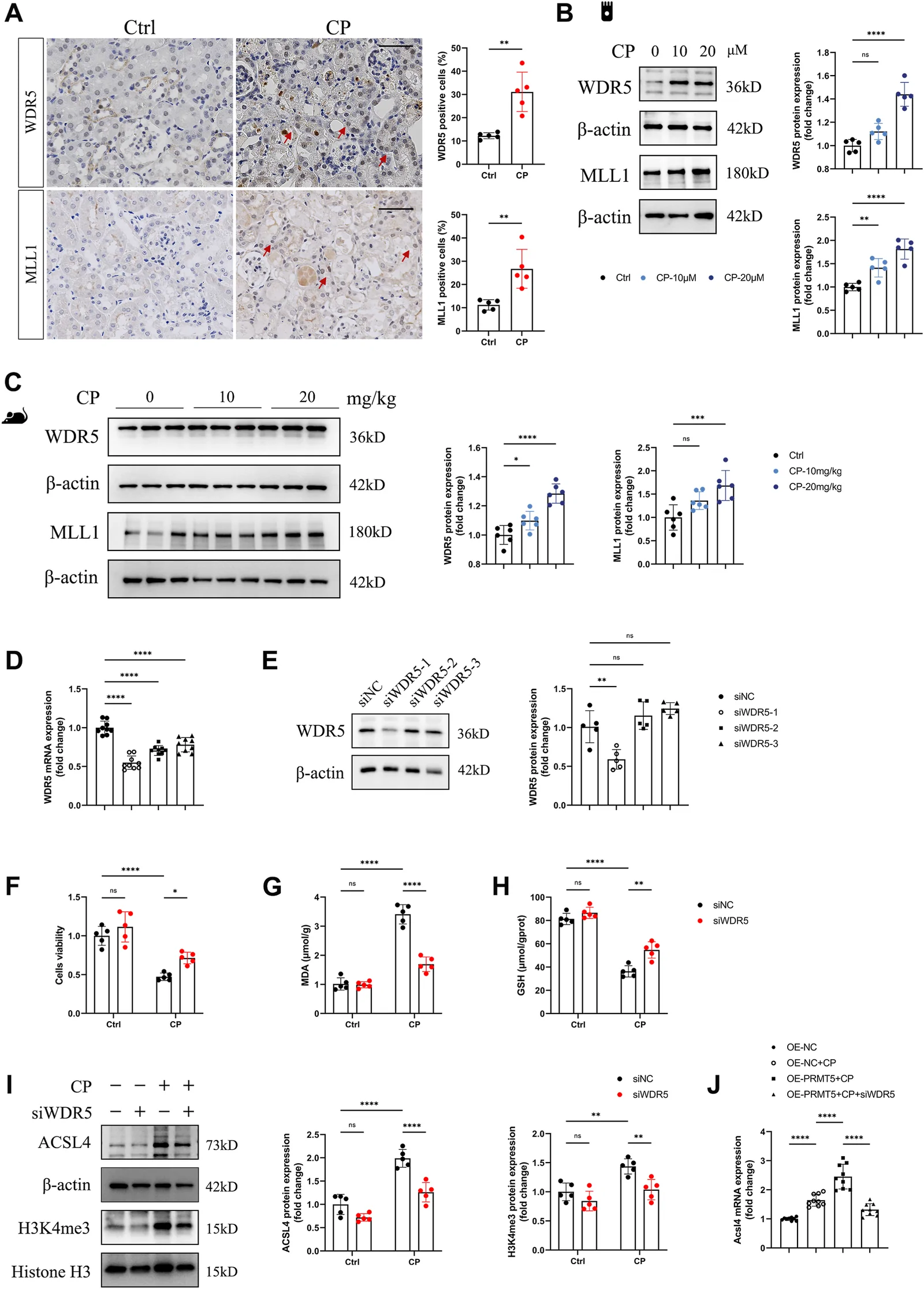
WDR5 is required to ACSL4-dependent tubular epithelial cell ferroptosis A. Representative IHC images and quantification of WDR5 and MLL1 expression in kidney from CP-AKI mice. Red arrows indicate positive stained tubular epithelial cells. Scale bar, 50 μm. B. Western blot analysis of WDR5 and MLL1 expression in HK-2 cells treated with cisplatin. C. Western blot analysis of WDR5 and MLL1 expression from CP-AKI mice kidney tissue. (D-I) HK-2 cells were transfected with siWDR5 prior to 20 μM cisplatin treatment.D. Relative WDR5 mRNA expression.E. Western blot analysis and quantification of WDR5.F. Assessment of cell viability after WDR5 knockdown.G. MDA content.H. GSH levels in HK-2 cells.I. Western blot analysis of ACSL4 and H3K4me3 in WDR5-knockdown HK-2 cells.J. Relative ACSL4 mRNA expression following PRMT5 overexpression and WDR5 knockdown. Data are presented as mean ± SD, n = 5-6 independent biological experiments. Technical replicates were averaged for analysis. Significance was assessed by one-way ANOVA with Tukey's post-hoc test or unpaired Student's *t*-test. NS, indicates no significant, *P < 0.05, **P < 0.01, ***P < 0.005, ****P < 0.001.

### 3.6 Pharmacological targeting and prophylactic intervention of PRMT5 attenuated experimental AKI

To evaluate the therapeutic potential of targeting PRMT5, the highly selective small molecule inhibitor GSK591 was administered to mice following cisplatin challenge (Fig. 8A). Mice treated with GSK591 exhibited the preservation of kidney weight (Fig. 8B) and a significant improvement of renal dysfunction (Fig. 8C). Histological analysis via H&E and PAS staining showed that GSK591-treated mice had lower histopathology injury scores, with fewer necrotic tubules and casts compared to the vehicle-treated CP-AKI group (Fig. 8D). Furthermore, TUNEL and NGAL staining confirmed that pharmacological inhibition of PRMT5 significantly decreased apoptotic cell death and suppressed the induction of the tubular injury marker NGAL (Fig. 8D). Given the regulatory role of PRMT5 on ferroptosis, we next evaluated the expression of ferroptosis-related proteins in the kidney tissue slices. Cisplatin-induced upregulation of the pro-ferroptotic driver ACSL4 was substantially suppressed by GSK591 treatment. Concurrently, the downregulation of NRF2 and FTH was markedly restored in the GSK591-treated group (Fig. 8E). These results suggest that pharmacological intervention targeting PRMT5 with GSK591 mitigates tubular injury and lipid peroxidation, supporting PRMT5 as a potential target in experimental renal damage.

**Fig. 8:**
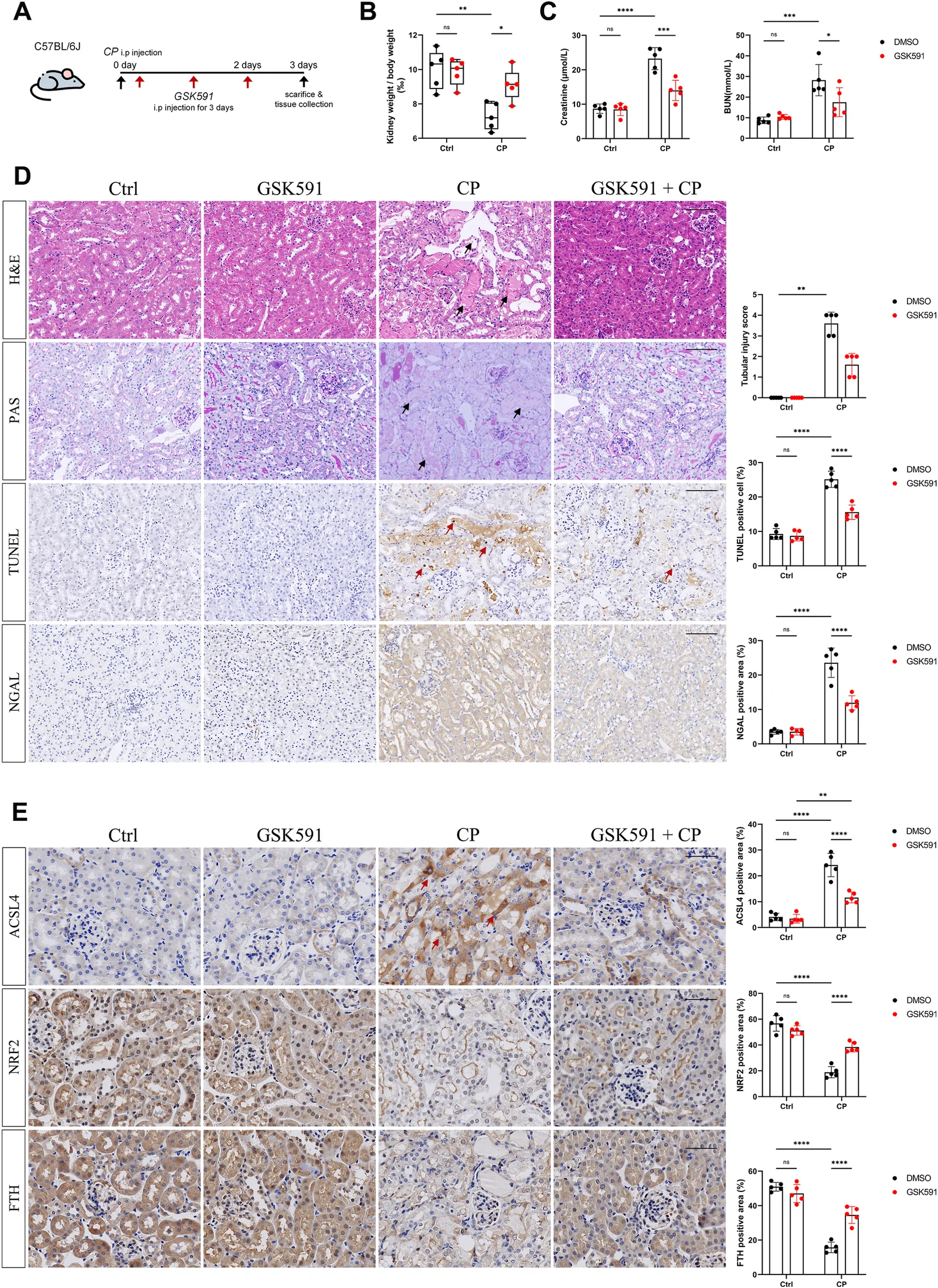
GSK591 administration improves renal function and attenuates tubular injury in CP-AKI. A. Schematic of the experimental design. Mice received a single dose of cisplatin followed by i.p. injection of GSK591 (a specific PRMT5 inhibitor) for 3 consecutive days. B. Kidney to body weight ratio in CP-AKI mice (9.112 ± 0.912‰ in the GSK591 + CP group vs. 7.279 ± 0.790‰ in the CP group). C. SCr and BUN level. D. Representative images of H&E, PAS, TUNEL and NGAL staining show tubular injury in kidneys. Right panels show the quantification analysis. Black arrows indicate tubular dilation, cast formation and epithelial shedding. Red arrows indicate NGAL positive damaged tubules. Scale bars, 100 μm. E. IHC images and quantification of ferroptosis markers, including ACSL4, NRF2 and FTH. Red arrows indicate ACSL4 positive tubular cells. Scale bars, 50 μm. Data are presented as mean ± SD, n = 5 biological replicates in each group. Significance was assessed by one-way ANOVA with Tukey's post-hoc test, or Kruskal-Wallis test with Dunn's post-hoc test for the tubular injury scores. NS, indicates no significant, *P < 0.05, **P < 0.01, ***P < 0.005, ****P < 0.001.

To explore potential translational tools, cloperastine hydrochloride (Clo) was selected for preliminary evaluation. Clo was previously screened from FDA/EMA compound libraries through PRMT5-specific AlphaLISA assay and validated as the top hit to inhibit arginine methylation. Moreover, molecular docking predicted that Clo might interferes with the SAM-binding site of PRMT5 [35]. Therefore, the prophylactic efficacy of Clo was evaluated in CP-AKI mice (Fig. 9A). Serum biochemical and physical analysis showed that Clo administration caused a significant, dose-dependent reduction in kidney weight loss (Fig. 9B) as well as SCr and BUN levels (Fig. 9C), preserving renal function under cisplatin stress. H&E, PAS, and TUNEL staining were performed to assess the histopathological alteration. Clo-treated mice groups displayed well-preserved tubular structures with minimal cast formation, reduced epithelial cell apoptosis, and lower injury scores compared to the vehicle-treated CP-AKI group (Fig. 9D). Moreover, Western blot analysis confirmed that Clo administration robustly inhibited the induction of NGAL and suppressed global SDMA modification, suggesting its potent interference with the PRMT5 catalytic program (Fig. 9E). Given that PRMT5 associated ferroptosis, lipid peroxidation and ferroptotic markers were further examined. Clo treatment significantly decreased the accumulation of MDA (Fig. 9F) and restored GSH concentrations (Fig. 9G) in a dose-dependent manner. Furthermore, Western blot analysis demonstrated that Clo treatment remarkably downregulated the protein expression of the pro-ferroptotic driver ACSL4 (Fig. 9H). Collectively, these results indicate that cloperastine hydrochloride attenuates lipid peroxidation and ACSL4 expression alongside tubular injury in a prophylactic setting, suggesting its potential utility as a small molecule tool against cisplatin induced renal damage.

**Fig. 9:**
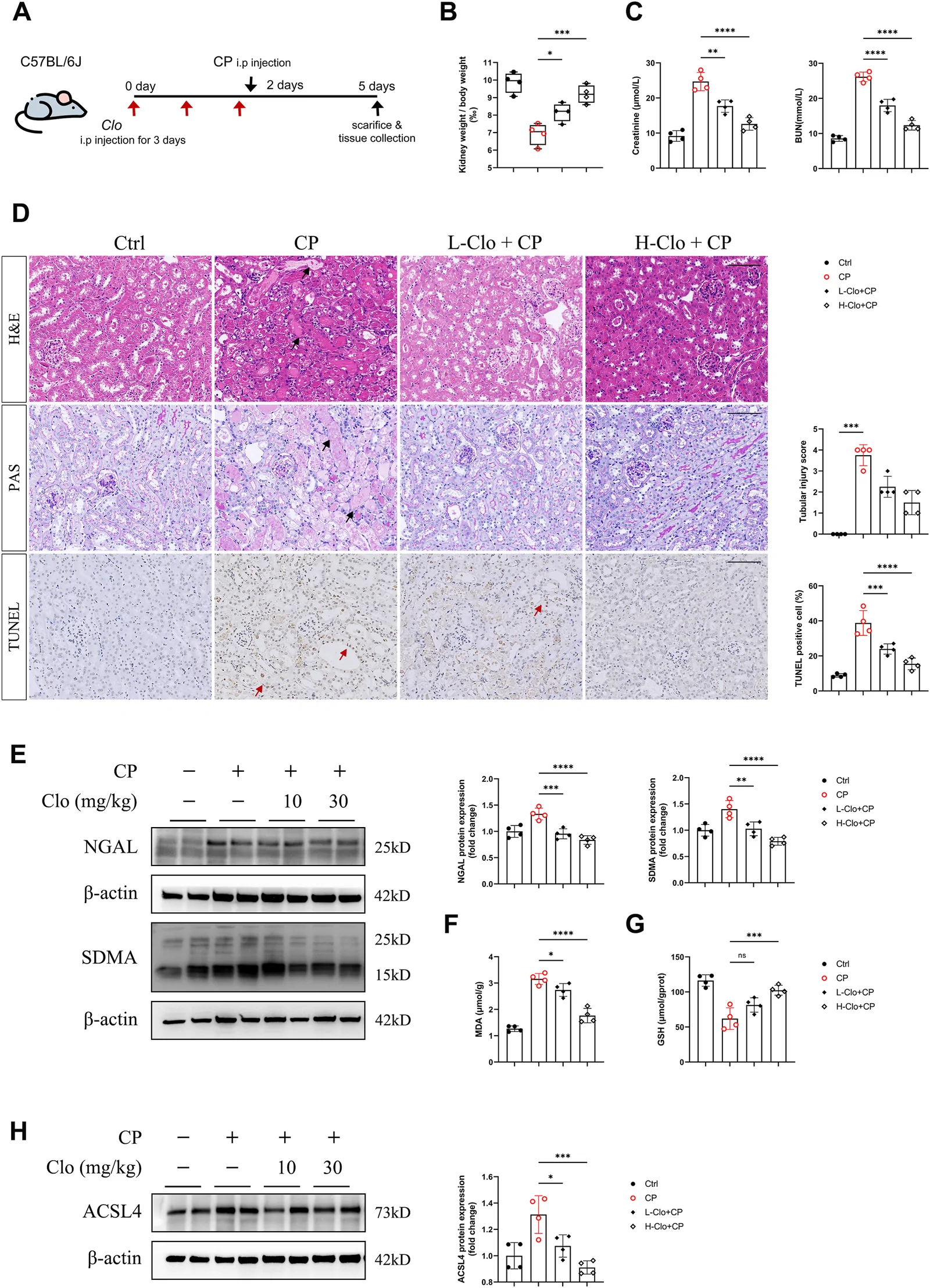
Drug repurposing identifies cloperastine hydrochloride as a potent strategy for PRMT5 inhibition in CP-AKI. A. Schematic of the experimental design. Pretreatment with cloperastine hydrochloride (Clo) by i.p. injection for 3 days prior to CP-AKI induction. The treatments included Clo at two doses: L-Clo (10 mg/kg) and H-Clo (30 mg/kg) B. Kidney to body weight ratio in CP-AKI mice (L-Clo + CP group: 8.161 ± 0.518‰, H-Clo + CP group: 9.196 ± 0.511‰, CP group: 6.926 ± 0.612‰).C. SCr and BUN level. D. Representative images of H&E and PAS staining showed tubular injury in kidneys, and histopathology score of tubular damage. Black arrow indicate tubular dilation, cast formation, loss of brush border and epithelial shedding. Red arrows indicate TUNEL stained in the renal tubules. Scale bars, 100 μm. E. Western blot analysis and quantification of NGAL and SDMA modification. F. MDA content of kidney tissues. G. GSH concentration of kidney tissues. H. Western blot analysis and quantification the protein expression of ACSL4. Data are presented as mean ± SD, n = 4 biological replicates in each group. Significance was assessed by one-way ANOVA with Tukey's post-hoc test, or Kruskal-Wallis test with Dunn's post-hoc test for the tubular injury scores. NS, indicates no significant, #p < 0.05, ##p < 0.01, ###p < 0.005, ####p < 0.001 vs the CP group.

## 4 Discussion

This study identified PRMT5 as a key epigenetic regulator of ACSL4 dependent ferroptosis in CP-AKI. Mechanistically, PRMT5 drove lipid peroxidation by activating ACSL4 transcription through the PRMT5/WDR5/H3K4me3 pathway. Crucially, pharmacological blockade of PRMT5 with its specific inhibitor GSK591 effectively mitigates renal injury in mice. Furthermore, prophylactic treatment with cloperastine suppressed the PRMT5-SDMA axis and mitigated tubular damage, supporting its preclinical protective effect.

The pathogenesis of AKI involves a complex interplay of hemodynamic changes [36], inflammation [37,38], and programmed cell death [39,40]. While much research focus has been placed on downstream signaling cascades, the upstream epigenetic mechanisms remain poorly understood [41,42]. Hence, identifying the epigenetic regulator responsible for transcription in AKI is important to understand the pathology. PRMTs regulates arginine methylation to modulate chromatin structure, transcription, and cell signaling. In recent research, the dysregulation of PRMTs contributes to inflammation, oxidative stress, and tubular injury in the kidney disease [43]. While PRMT1 promoted renal fibrosis through TGF-β1/Smad3 and Stat3/αVβ6 signaling [44,45], our findings indicated that PRMT5 plays a more prominent role in the acute injury phase. This expression pattern appears to differ from other PRMT family members. In chronic diabetic nephropathy (DN), CARM1 (PRMT4) degradation led to apoptosis through Notch1 pathway [46]. Conversely, Prmt6 has been identified as a driver of ferroptosis in DN by modulating Stat1/ACSL4 axis [47]. In contrast, the PRMT5-MEP50 complex was significantly and consistently upregulated across human AKI and three distinct murine models of acute stimulation. As a primary Type II methyltransferase, PRMT5 has been implicated in cellular responses to stress and DNA damage [48]. Toxic insults could induce acute DNA damage and other forms of cellular stress, which may increase the need for chromatin and transcriptional adaptation involving the PRMT5-MEP50 complex. The correlation between PRMT5 levels and SCr suggests that this complex may be involved in the tubular stress response to acute stress rather than a passive marker of filtration failure.

CP-AKI is associated with cell death processes, including apoptosis, necrosis, pyroptosis, and autophagy [40]. However, recent evidence suggests that ferroptosis has emerged as an important contributor of tubular injury, a regulated form of iron-dependent lipid peroxidation, is the more potent driver of tubular loss in AKI. Given the elevation on PRMT5 in AKI models, we hypothesized that it serves as an important epigenetic regulator of cisplatin-induced renal damage by promoting the ferroptotic pathway. In our assays, both Fer-1 and Nec-1 protected against PRMT5 overexpression induced cell death, suggesting that necroptosis and ferroptosis may co-exist as parallel regulated cell death pathway during acute kidney injury. However, Fer-1 provided the most effective rescue. This observation suggested that while cisplatin toxicity triggers multiple cell death programs, PRMT5 mediated injury preferentially drives lipid peroxidation as a predominant damage modality. In addition, ACSL4 dependent lipid peroxidation and associated ROS generation directly damage cell membranes. These oxidative events can also serve as upstream triggers to activate the RIPK1-RIPK3-MLKL pathway of necroptosis [49]. Therefore, we chose to focus on the role of PRMT5 in driving lipid peroxidation. The ferroptotic mechanism involves both the initiation and the defense of oxidative stress [50]. First, ACSL4 facilitates lipid metabolic remodeling, increasing the abundance of PUFAs in the tubular epithelial cell membrane, providing the necessary substrates for lipid hydroperoxide accumulation [51]. Our results indicated that PRMT5 depletion mitigated ACSL4-mediated lipid peroxidation in CP-AKI. Second, intracellular iron overload is indispensable for catalyzing the Fenton reaction that drives this lipid peroxidation [52]. Our findings demonstrated that PRMT5 knockdown significantly reduced the intracellular labile Fe^2+^ pool, blunted the upregulation of the iron transporter TFRC, and prevented the depletion of the storage protein FTH, remodeling of iron homeostasis prevents the accumulation of free iron under toxic stress. Third, antioxidant defense is damaged while ferroptosis occurrence in AKI [53]. It was evidenced that PRMT5 contributed to a depletion of GSH in CP-AKI model. In summary, these findings showed that PRMT5 promoted cell death by triggering by altering lipid substrates, iron metabolic disorders and antioxidant defense failure.

The induction of PRMT5 appears to drive an epigenetic program that promotes ferroptotic cell death. In particular, the potential cross-talk between PRMT5 mediated SDMA deposition and WDR5 associated chromatin remodeling is noteworthy [[54], [55], [56]]. As a core scaffold for the MLL1 (Mixed-Lineage Leukemia) complex, WDR5 is required for the stability and catalytic efficiency of H3K4 methyltransferase. Its recruitment drives the enrichment of H3K4me3, a mark of transcriptional activation, at target gene promoters [34,57,58]. We reasoned that PRMT5 might promote WDR5 mediated H3K4me3 enrichment to activate pro-ferroptosis genes. To assess this hypothesis, we first examined the global histone marks. Western blot analysis showed that cisplatin treatment increased global H3R2me2s and H3K4me3 levels, while PRMT5 knockdown reversed these increases. CUT&Tag profiling revealed that cisplatin broadly enhanced H3K4me3 signal intensity at gene promoter regions. At the *ACSL4* locus specifically, CUT&RUN-qPCR analysis showed that cisplatin promoted H3R2me2s deposition, WDR5 binding, and H3K4me3 enrichment. This local epigenetic modification was associated with chromatin relaxation, enabling transcriptional activation of *ACSL4*. Conversely, PRMT5 silencing significantly reduced these modifications and suppressed *ACSL4* transcription. We acknowledge that methylarginine antibodies sometimes show cross-reactivity. However, PRMT5 silencing led to a consistent decrease in multiple symmetric di-methylation marks, including H3R2me2s, H3R8me2s, and H4R3me2s. These multi-target data strongly validate the specificity of our epigenetic findings. Importantly, WDR5 knockdown reversed the upregulation of ACSL4 and protected tubular cells from lipid peroxidation. Thus, PRMT5 functions as an epigenetic activator of the pro-ferroptotic *ACSL4* transcription.

It is critical to note that the role of PRMT5 in ferroptosis appears to be disease context-dependent. Recent tumor studies showed that PRMT5 suppresses ferroptosis in cancer cells by non-histone protein methylation such as ACSL4 and GPX4 [59,60]. In contrast, our study revealed that PRMT5 acts as a pro-ferroptotic driver in CP-AKI models. This discrepancy primarily stems from the fundamental differences between tumor biology and acute kidney injury. Under acute nephrotoxic stress, PRMT5 in tubular cells predominantly functions as an epigenetic regulator. Instead of focusing on post-translational protein stability, our results demonstrated that PRMT5 modulates histone modifications in the nucleus to regulate ferroptosis related genes transcriptions. Therefore, the distinct microenvironment of AKI shifts the regulatory priority of PRMT5 toward epigenetic histone modification, leading to an opposite pathological outcome.

A major challenge in nephrology is preventing the progression from AKI to CKD. Incomplete repair after AKI often leads to persistent fibrosis [61]. Ferroptosis has recently been implicated as a driver of this transition. The release of damage-associated molecular patterns (DAMPs) from ferroptotic cells could amplify renal inflammation, triggering myofibroblast activation and chronic interstitial fibrosis [62]. In our study, PRMT5 knockdown reduced the filtration of F4/80^+^ macrophages and Ly6G^+^ neutrophils. These findings showed that inhibiting ferroptosis would break the necroinflammatory feedback loop. In addition, PRMT5 deficiency increased EdU incorporation, indicating enhanced tubular adaptive repair. In short, these acute-phase findings hint at a possible role of PRMT5 in early tissue remodeling. However, since this study primarily focused on the acute phase of cisplatin injury, whether these cellular changes directly alter long-term maladaptive repair or chronic interstitial fibrosis requires further investigation using established AKI to CKD models.

To evaluate the effect of targeting PRMT5 during renal injury, a highly potent and selective small-molecule inhibitor, GSK591 was administered in CP-AKI mice treatment. Post-treatment with GSK591 restored renal function, and effectively suppress lipid peroxidation. Cloperastine hydrochloride (Clo), an established antitussive, has been identified as a potential PRMT5 inhibitor [63]. In our AKI model, prophylactic Clo administration suppressed the PRMT5-SDMA axis, restored renal function, and alleviated pathological damage during cisplatin-induced injury. Although Clo has an established clinical safety profile [64], its application as a protective strategy against nephrotoxic insults remains preliminary and requires cautious evaluation. As a centrally acting drug, Clo can cross the blood-brain barrier, and may cause central nervous system (CNS) effects, including somnolence or sedation [63]. In our murine model, no obvious behavioral changes were observed. However, Clo undergoes hepatic metabolism and is eliminated through both renal and biliary routes. These findings do not exclude the possibility of CNS effects under conditions of renal impairment. Impaired renal clearance during AKI may alter Clo exposure and increase the risk of CNS toxicity. Therefore, further studies are needed to assess its pharmacokinetic and safety in renal injury models, as well as the optimal dose and treatment window. Structural modifications or kidney-targeted delivery approaches may also help reduce unnecessary CNS exposure [65].

Several limitations should be acknowledged. First, our current findings highlight a functional association between PRMT5 and *ACSL4* promoter modifications. Beyond its primary methyltransferase activity, PRMT5 may also participate in broader chromatin complexes, and the precise enzymatic contribution versus scaffolding roles will require further studies. Second, human control kidney tissues were restricted to para-carcinoma samples due to ethical constraints, and the potential crosstalk between PRMT5 and other regulated cell death modalities, such as necroptosis, requires further study. Finally, longitudinal animal studies are needed to evaluate whether PRMT5 inhibition mitigates long-term transition to chronic kidney disease.

## 5 Conclusion

In summary, this study identified PRMT5 as an important upstream epigenetic contributor to ACSL4-driven ferroptosis in CP-AKI. Through WDR5 associated H3K4me3 enrichment at the *ACSL4* promoter, PRMT5 promoted ACSL4 expression, leading to lipid peroxidation that caused tubular injury. Pharmacological inhibition of PRMT5 with GSK591 supported the functional relevance of this target in kidney injury. Notably, prophylactic treatment with the small molecule inhibitor cloperastine hydrochloride reduced PRMT5 associated lipid peroxidation and tubular injury in experimental models, highlighting a plausible candidate for further exploration. These findings indicate that PRMT5/WDR5/H3K4me3 axis is associated with ACSL4 driven ferroptosis and provide a useful foundation for developing novel epigenetic interventions against nephrotoxic insults.

## CRediT authorship contribution statement

**Jiawen Lin:** Writing – review & editing, Writing – original draft, Validation, Project administration, Investigation, Formal analysis, Data curation. **Junxuan Fang:** Writing – review & editing, Formal analysis. **Tianjiao Cui:** Writing – review & editing, Investigation. **Jiaqi Xu:** Methodology, Investigation. **Wenjian Lin:** Investigation. **Shuping Li:** Methodology. **Yuan Sui:** Methodology. **Zhihua Zheng:** Supervision, Funding acquisition. **Keping Wu:** Writing – review & editing, Supervision. **Mingcheng Huang:** Writing – review & editing, Supervision, Conceptualization.

## Ethics declaration

Written informed consent to take part in the study and to publish the article has been obtained from all participants or their legal representatives. The privacy rights of participants have been observed.

This study included organ or tissue donors. This study includes human biological material and consent was obtained by donors, or their next of kin or legal representatives, for use in this study and for publication of the article. The samples used in this research were not sourced from executed prisoners or prisoners of conscience.

This study was performed in compliance with relevant laws, regulatory frameworks and guidelines where the research took place. This study was approved by the Seventh Affiliated Hospital, Sun Yat-Sen University Ethics Committee. (Approval No. KY-2026-015-01)

This study was conducted in accordance with the ARRIVE (Animal Research: Reporting of In Vivo Experiments) guidelines. This study was approved by the Sun Yat-Sen University The Institutional Animal Care and Use Committee. (Approval No. SYSU-IACUC-2024-002652)

## Funding sources

This work was supported by 10.13039/501100012151Sanming Project of Medicine in Shenzhen (Grant No. SZSM202411016), 10.13039/501100010877Science, Technology and Innovation Commission of Shenzhen Municipality (Grant No. JCYJ20250604143421028), and the 10.13039/501100021171Guangdong Basic and Applied Basic Research Foundation of China (Grant No. 2023A1515111043).

## Declaration of competing interest

The authors declare that they have no known competing financial interests or personal relationships that could have appeared to influence the work reported in this paper.

## Data Availability

Data will be made available on request.

## Abbreviations

AKI: acute kidney injury
CKD: chronic kidney disease
ESRD: end stage renal disease
CP: cisplatin
CP-AKI: cisplatin-induced acute kidney injury
S-AKI: sepsis-associated acute kidney injury
FA-AKI: folic acid induced acute kidney injury
LPS: lipopolysaccharide
TEC: proximal tubular epithelial cell
PRMT5: protein methyltransferase 5
SDMA: symmetric di-methylation of arginine
MEP50: methylosome protein 50
WDR5: WD repeat domain 5
MLL1: Myeloid/lymphoid or mixed-lineage leukemia
HK-2 cell: human renal tubular epithelial cell
NGAL: neutrophil gelatinase-associated lipocalin
ACSL4: Acyl-CoA synthetase long-chain family member 4
LPCAT3: Lysophosphatidylcholine acyltransferase 3
4HNE: 4-hydroxynonenal
GPX4: glutathione peroxidase 4
NRF2: Nuclear factor erythroid 2 related factor 2
KEAP1: Kelch-like ECH associated protein 1
xCT: Cystine/glutamate transporter
TFRC: transferrin receptor
FTH: ferritin heavy chain
MDA: malondialdehyde
GSH: glutathione
GSSG: oxidized glutathione
SCr: serum creatinine
BUN: blood urea nitrogen
AAV: Adeno-associated Virus
RSL3: RAS selective lethal 3
Fer-1: Ferrostatin-1
Nec-1: Necrostatin-1
3-MA: 3-methyladenine
Clo: cloperastine hydrochloride
CUT&TAG: Cleavage Under Targets and Tagmentation
CUT&RUN: Cleavage Under Targets and Release Using Nuclease
TEM: transmission electron microscopy
