# Supplementary material for: PRMT5 promotes ferroptosis in cisplatin-induced acute kidney injury through WDR5/ACSL4 pathway
Source: Redox Biol. 2026 Oct 1;97:104421. doi: 10.1016/j.redox.2026.104421 (PMC13659685; doi:10.1016/j.redox.2026.104421)
Supplement: Supplementary file 1 [file mmc1.docx]

Supplementary Materials and Methods

Supplementary Figure 1. Expression of PRMT5 in tubular epithelial cells under AKI inducing conditions.

Supplementary Figure 2. Expression profile of PRMT family in tubular epithelial cells.

Supplementary Figure 3. Nuclear localization of PRMT5 and SDMA is enhanced by cisplatin in HK-2 cells.

Supplementary Figure 4. PRMT5 regulated the expression of ferroptosis related genes in cisplatin treated HK-2 cells.

Supplementary Figure 5. PRMT5 knockdown decreased lipid peroxidation in mice kidney tissues.

Supplementary Figure 6. RNA-sequencing analysis reveals PRMTs as a key transcriptional regulator in cisplatin-induced HK-2 cells.

Supplementary Figure 7. PRMT5 activated epithelial tubular cells histone modification after cisplatin exposure.

Supplementary Table 1. Clinical information of AKI and control patients

Supplementary Table 2. siRNA and lentiviral sequences used in cell experiment

Supplementary Table 3. Summary of pharmacological agents and treatment regimens in experiments

Supplementary Table 4. AAV sequences used in mice experiment

Supplementary Table 5. Reagents used in the study

Supplementary Table 6. Antibodies used in the study

Supplementary Table 7. Primer sequences in RT-qPCR

Supplementary Table 8. Primer sequences in CUT&RUN-qPCR

**Supplemental Materials and Methods**

**Cell Culture and Treatment**

The rat renal proximal tubular cell line NRK-52E were cultured in DMEM supplemented with 10% FBS and 1% penicillin/streptomycin in a humidified incubator at 37°C with 5% CO_2_. To establish the in vitro AKI model, NRK-52E cells were seeded at an appropriate density and allowed to reach 80% confluence and then exposed to cisplatin at concentrations of 20 μM for 24 h to induce injury.

**Bioinformatic Analysis of Public Datasets**

Public RNA-sequencing datasets (GSE165100, GSE227623, and GSE87024) were downloaded from the NCBI Gene Expression Omnibus (GEO) database to evaluate the expression profiles of the PRMT family. Expression matrices for mice kidney tissue under cisplatin, LPS and IRI treatment were extracted. Gene ID conversion was performed using the org.Mm.eg.db R package. Normalization and differential expression analysis between control and model groups were conducted using the limma R package to calculate log_2_FC and P-value for each PRMT family member, with statistical significance set at *P* < 0.05. Individual sample expression levels for all PRMT family members were visualized as heatmap using the pheatmap R package. To comprehensively present the statistical significance and magnitude of alteration across models, the corresponding log_2_FC and -log10 (P-value) for each gene were integrated and displayed as dot plots alongside the heatmap using ggplot2.

**Supplemental Figures**

**Supplementary Figure 1. Expression of PRMT5 in tubular epithelial cells under AKI inducing conditions. (**A-D) C57BL/6 mice were intraperitoneal injection of 20mg/kg LPS, n=5 biological replicates in each group. (A) SCr and BUN levels in LPS induced sepsis associated AKI (S-AKI) mice. (B) Representative images of H&E staining showed tubular injury in kidneys, and histopathology score of tubular damage. Black arrows indicate tubular dilation, loss of brush border and epithelial shedding. Scale bars, 200 μm. (C) Western blot analysis and quantification assessment of PRMT5 expression. (D) Immunohistochemistry analysis and quantification of PRMT5. Red arrows indicate the epithelial cells stained for PRMT5. Scale bars, 200 μm. (E-H) C57BL/6mice were intraperitoneal injection of 250mg/kg folic acid, n=5 biological replicates in each group. (E) SCr and BUN levels in folic acid induced AKI (FA-AKI) mice. (F) Representative images of H&E staining showed tubular injury in kidneys, and histopathology score of tubular damage. Black arrows indicate tubular dilation, cast formation, loss of brush border and epithelial shedding. Scale bars, 200 μm. (G) Western blot analysis and quantification assessment of PRMT5 expression. (H) Immunohistochemistry analysis and quantification of PRMT5. Red arrows indicate the epithelial cells stained for PRMT5. Scale bars, 200 μm. (I) Western blot analysis and quantification of PRMT5, MEP50 and SDMA in NRK cells treated with 20 μM cisplatin, n=3 independent biological experiments. Data are presented as mean ± SD. Significance was assessed by unpaired Student *t*-test, or Mann-Whiney test for tubular injury scores. NS, indicates no significant, *p<0.05, **p<0.01, ***p<0.005, ****p<0.001.

**Supplementary Figure 2. Expression profile of PRMT family in tubular epithelial cells. (A)** Bioinformatic screening. Heatmap showing mRNA levels of PRMT family members in mice kidney tissue from public datasets (GSE165100, GSE227623, and GSE87024). **(B)** RT-qPCR validation. The mRNA levels of PRMT members in HK-2 cells exposed to 20 μM cisplatin, 10 μg/ml LPS, or hypoxia/reoxygenation (H/R). For the H/R model, cells were subjected to 8 h of hypoxia/glucose deprivation followed by 16 h of reoxygenation. Data are presented as mean ± SD (three biological samples per group, each with technical triplicates). Significance was assessed by unpaired Student *t*-test. NS, indicates no significant, *p<0.05, **p<0.01, ***p<0.005, ****p<0.001.

**Supplementary Figure 3. Nuclear localization of PRMT5 and SDMA is enhanced by cisplatin in HK-2 cells.** Representative immunofluorescence images of PRMT5 (A) and SDMA (B) stained HK-2 cells. White arrows indicate the increased nuclear localization of PRMT5 and SDMA marks in cisplatin treated cells. Nuclei were counterstained with DAPI (blue). Scale bars, 3μm for (A) high magnification and 50μm for (B) low magnification. Data are presented as mean ± SD, n=5 independent biological experiments. Significance was assessed by unpaired Student *t*-test. ***p<0.005, **p<0.001.

**Supplementary Figure 4. PRMT5 regulated the expression of ferroptosis related genes in cisplatin treated HK-2 cells. (A-F)** The mRNA expression profiles of key ferroptosis related genes were analyzed by RT-qPCR. Data are presented as mean ± SD, n=5 independent biological experiments. Technical replicates were averaged for analysis. Significance was assessed by one-way ANOVA with Tukey’s post-hoc test. NS, indicates no significant, *p<0.05, **p<0.01, ***p<0.005, ****p<0.001. NS indicates not significant.

**Supplementary Figure 5. PRMT5 knockdown decreased lipid peroxidation in mice kidney tissues.** (A) Representative IHC staining images and quantification of 4HNE and ACSL4 in mice kidney slices. n = 6 biological replicates in each group. Scale bars, 50 μm. (B) Representative immunofluorescence images of 4-HNE expression (red) in mouse kidney tissues. Nuclei were counterstained with DAPI (blue). Scale bar, 100 µm. Data are presented as mean ± SD. Significance was assessed by one-way ANOVA with Tukey’s post-hoc test. NS, indicates no significant, **P*<0.05, ***P*<0.01, ****P*<0.005, *****P*<0.001. NS indicates not significant.

**Supplementary Figure 6. RNA-sequencing analysis reveals PRMTs as a key transcriptional regulator in cisplatin-induced HK-2 cells.** (A) Principal component analysis (PCA) plot demonstrating distinct clustering of gene expression profiles between CP treatment and siPRMT5+CP treatment group. (B) Heatmap illustrating the differential expressed gens (DEGs) between the CP and siPRMT5+CP group. (C) Gene ontology (GO) enrichment analysis of the DEGs identified key biological processes (BP), molecular functions (MF) and cellular components (CC) primarily regulated by PMT5 in cisplatin-induced HK-2 cells injury. This analysis suggested PRMT5 was involvement in broad transcriptional regulatory networks.

**Supplementary Figure 7. PRMT5 activated epithelial tubular cells histone modification after cisplatin exposure.** (A) Western blot analysis of histone modifications from cisplatin induced HK-2 cells. Data are presented as mean ± SD, n=5 independent biological experiments. Significance was assessed by one-way ANOVA with Tukey’s post-hoc test. NS, indicates no significant, *p<0.05, **p<0.01, ***p<0.005, ****p<0.001. NS indicates not significant.

**Supplementary Table 1**. Clinical information of AKI and control patients

| **Patient ID** | **Gender** | **Age** | **BUN**  **(mmol/L)** | **SCr**  **(μmol/L)** | **eGFR**  **(ml/min/1.7m^2^)** | **AKI**  **Etiology** | **Biopsy**  **indication** |
| --- | --- | --- | --- | --- | --- | --- | --- |
| 1 | Female | 67 | 22.8 | 219.0 | 22 | Drug-induced tubular injury | Unexplained AKI post-platinum |
| 2 | Female | 21 | 5.2 | 147 | 44 | Drug-induced tubular injury | Unexplained AKI post-platinum |
| 3 | Male | 34 | 11.1 | 156.6 | 45 | MCD with acute tubular injury | rapid edema and acute oliguria |
| 4 | Female | 42 | 12.6 | 179.2 | 31 | IgA nephropathy with acute tubulointerstitial nephritis | Acute renal dysfunction with hematuria and edema |
| 5 | Male | 72 | 12.4 | 123.9 | 53 | Drug-induced  ATN | Unexplained AKI after NSAID with melena |
| **Patient ID** | **Gender** | **Age** | **BUN**  **(mmol/L)** | **SCr**  **(μmol/L)** | **eGFR**  **(ml/min/1.7m^2^)** | **Control subjects** | |
| 6 | Female | 33 | 3.7 | 54 | 117 | Para-carcinoma renal tissues from patients undergoing nephrectomy | |
| 7 | Female | 26 | 3.3 | 49.6 | 127 |  |  |
| 8 | Male | 49 | 6.6 | 74.1 | 113 |  |  |
| 9 | Male | 66 | 6.5 | 81.9 | 94 |  |  |
| 10 | Male | 58 | 4.6 | 84.3 | 101 |  |  |

**Supplementary Table 2**. siRNA, lentiviral and sequences in the study

| **Gene** | **Sequence 5’-3’** | **Species** |
| --- | --- | --- |
| siPRMT5 -1 | UCAAGAACUCCCUGGAAUATT | Human |
| siPRMT5 -2 | CCAGUUUGAGAUGCCUUAUTT |  |
| siPRMT5 -3 | GGACCTGAGAGATGATATA |  |
| siWDR5 -1 | GCUCAGAGGAUAACCUUGU |  |
| siWDR5 -2 | GGAAGUGCCUCAAGACUUU |  |
| siWDR5 -3 | CAGAGGAUAACCUUGUUUA |  |
| PRMT5  overexpressing lentivirus | ATGGCGGCGATGGCGGTCGGGGGTGCTGGTGGGAGCCGCGTGTCCAGCGGGAGGGACCTGAATTGCGTCCCCGAAATAGCTGACACACTAGGGGCTGTGGCCAAGCAGGGGTTTGATTTCCTCTGCATGCCTGTCTTCCATCCGCGTTTCAAGAGGGAGTTCATTCAGGAACCTGCTAAGAATCGGCCCGGTCCCCAGACACGATCAGACCTACTGCTGTCAGGAAGGGACTGGAATACGTTAATTGTGGGAAAGCTTTCTCCATGGATTCGTCCAGACTCAAAAGTGGAGAAGATTCGCAGGAACTCCGAGGCGGCCATGTTACAGGAGCTGAATTTTGGTGCATATTTGGGTCTTCCAGCTTTCCTGCTGCCCCTTAATCAGGAAGATAACACCAACCTGGCCAGAGTTTTGACCAACCACATCCATACTGGCCATCACTCTTCCATGTTCTGGATGCGGGTACCCTTGGTGGCACCAGAGGACCTGAGAGATGATATAATTGAGAATGCACCAACTACACACACAGAGGAGTACAGTGGGGAGGAGAAAACGTGGATGTGGTGGCACAACTTCCGGACTTTGTGTGACTATAGTAAGAGGATTGCAGTGGCTCTTGAAATTGGGGCTGACCTCCCATCTAATCATGTCATTGATCGCTGGCTTGGGGAGCCCATCAAAGCAGCCATTCTCCCCACTAGCATTTTCCTGACCAATAAGAAGGGATTTCCTGTTCTTTCTAAGATGCACCAGAGGCTCATCTTCCGGCTCCTCAAGTTGGAGGTGCAGTTCATCATCACAGGCACCAACCACCACTCAGAGAAGGAGTTCTGCTCCTACCTCCAATACCTGGAATACTTAAGCCAGAACCGTCCTCCACCTAATGCCTATGAACTCTTTGCCAAGGGCTATGAAGACTATCTGCAGTCCCCGCTTCAGCCACTGATGGACAATCTGGAATCTCAGACATATGAAGTGTTTGAAAAGGACCCCATCAAATACTCTCAGTACCAGCAGGCCATCTATAAATGTCTGCTAGACCGAGTACCAGAAGAGGAGAAGGATACCAATGTCCAGGTACTGATGGTGCTGGGAGCAGGACGGGGACCCCTGGTGAACGCTTCCCTGCGGGCAGCCAAGCAGGCCGACCGGCGGATAAAGCTGTATGCTGTGGAGAAAAACCCAAATGCCGTGGTGACGCTAGAGAACTGGCAGTTTGAAGAATGGGGAAGCCAAGTGACCGTAGTCTCATCAGACATGAGGGAATGGGTGGCTCCAGAGAAAGCAGACATCATTGTCAGTGAGCTTCTGGGCTCATTTGCTGACAATGAATTGTCGCCTGAGTGCCTGGATGGAGCCCAGCACTTCCTAAAAGATGATGGTGTGAGCATCCCCGGGGAGTACACTTCCTTTCTGGCTCCCATCTCTTCCTCCAAGCTGTACAATGAGGTCCGAGCCTGTAGGGAGAAGGACCGTGACCCTGAGGCCCAGTTTGAGATGCCTTATGTGGTACGGCTGCACAACTTCCACCAGCTCTCTGCACCCCAGCCCTGTTTCACCTTCAGCCATCCCAACAGAGATCCTATGATTGACAACAACCGCTATTGCACCTTGGAATTTCCTGTGGAGGTGAACACAGTACTACATGGCTTTGCCGGCTACTTTGAGACTGTGCTTTATCAGGACATCACTCTGAGTATCCGTCCAGAGACTCACTCTCCTGGGATGTTCTCATGGTTTCCCATCCTCTTCCCTATTAAGCAGCCCATAACGGTACGTGAAGGCCAAACCATCTGTGTGCGTTTCTGGCGATGCAGCAATTCCAAGAAGGTGTGGTATGAGTGGGCTGTGACAGCACCAGTCTGTTCTGCTATTCATAACCCCACAGGCCGCTCATATACCATTGGCCTCGGTATGGACTACAAGGATGACG |  |

**Supplementary Table 3**. Summary of pharmacological agents and treatment regimens in experiments

| **Experimental Stage** | **AKI inducer**  **& Dose** | **Treatment  Agent & Dose** | **Route** |
| --- | --- | --- | --- |
| Model validation | cisplatin  (10 or 20 mg/kg, i.p.) | None | Single dose,  harvested after injection 72 h |
|  | lipopolysaccharide  (20 mg/kg i.p.) | None | Single dose,  harvested after injection 24 h |
|  | folic acid  (250 mg/kg i.p.) | Noe | Single dose,  harvested after injection 48 h |
| Therapeutic  Intervention | cisplatin  ( 20 mg/kg, i.p.) | GSK591  (50mg/kg 1.p.) | Administered at 1 h, 24 h, and 48 h after CP injection, harvested after CP i.p. 72 h |
| Preventive  Intervention | cisplatin  (20 mg/kg, i.p.) | cloperastine hydrochloride  (10 or 30mg/kg i.p.) | Daily for 3 consecutive days before cisplatin injection,  harvested after CP i.p. 72 h |

**Supplementary Table 4**. AAV sequences used in mice experiment

| **Gene** | **Sequence 5’-3’** | **Species** |
| --- | --- | --- |
| AAV-GGT-shPRMT5 | GGACCTGAGAGATGATGTAAT | Mouse |
|  | ATTACATCATCTCTCAGGTCC |  |

**Supplementary Table 5**. Reagents used in the study

| **Reagents** | **Vendors** |
| --- | --- |
| Cisplatin | MedChemExpress |
| Lipopolysaccharide (LPS) | Sigma-Aldrich |
| Folic acid | MedChemExpress |
| GSK591 | MedChemExpress |
| Candesartan cilexetil | MedChemExpress |
| Cloperastine hydrochloride | MedChemExpress |
| Ferrostatin-1 (Fer-1) | MedChemExpress |
| Z-VAD-FMK | MedChemExpress |
| Necrosulfonamide (Nec-1) | MedChemExpress |
| 3-Methylamine (3-MA) | MedChemExpress |
| RSL3 | MedChemExpress |
| Erastin | MedChemExpress |
| Cycloheximide (CHX) | TargetMol |
| Transfection reagents | GenePharma |
| Lotus tetragonolobus lectin (LTL) | Vector Laboratories |
| CUT&TAG Library Kit | Vazyme |
| CUT&RUN-qPCR | Vazyme |
| C11-BODIPY | Dojindo |
| FerroOrange | Dojindo |
| Mito-FerroGreen | Dojindo |
| MitoSOX | Therm Fisher Scientific |
| One-Step TUNEL Assay Kit | Abbkine |
| DAB TUNEL Detection Kit | Servicebio |
| EDU Assay Kit | Beyotime |
| TRIzol | Vazyme |
| SYBR Green master mix | Vazyme |
| Reverse Transcription Kit | Vazyme |
| MDA Assay Kit | Beyotime |
| GSH/GSSG Assay Kit | Beyotime |
| ATP Assay Kit | Beyotime |
| NC membranes | Pall |

**Supplementary Table 6**. Antibodies used in the study

| **Antigen** | **Reference** | **Customer** |
| --- | --- | --- |
| Anti- PRMT5 | ab109451 | Abcam |
| Anti- MEP50 | 2018 | Cell signaling Technology |
| Anti- WDR5 | 13105 | Cell signaling Technology |
| Anti- MLL1 | 29278-1-AP | Proteintech |
| Anti- symmetric dimethylarginine (SDMA) | 13222 | Cell signaling Technology |
| Anti- NGAL | DF6818 | Affinity |
| Anti- ACSL4 | 22401-1-AP | Proteintech |
| Anti- 4 HNE | ab48506 | Abcam |
| Anti- GPX4 | ab125066 | Abcam |
| Anti- NRF2 | 16396-1-AP | Proteintech |
| Anti- xCT | 26864-1-AP | Proteintech |
| Anti- TFRC | A25900 | Abclonal |
| Anti- FTH | T55648 | Abmart |
| Anti- β-actin | 20536-1-AP | Proteintech |
| Anti- H3R2me2s | A2373 | Abclonal |
| Anti- H3K4me3 | DF6935 | Affinity |
| Anti- H3R8me2s | A-3706 | Epigentek |
| Anti- H4R3me2s | 61187 | Active Motif |
| Anti- Histone 3 | T56587 | Abmart |
| Goat Anti-Rabbit IgG | SA00001-2 | Proteintech |
| Goat Anti-Rabbit IgG-594 | SA00013-4 | Proteintech |

**Supplementary Table 7**. Primer sequences in RT-qPCR

| **Gene** | **Sequence 5’-3’** | | **Species** |
| --- | --- | --- | --- |
| PRMT1 | Forward: | TGCGGTGAAGATCGTCAAAGCC | Human |
|  | Reverse: | GGACTCGTAGAAGAGGCAGTAG |  |
| PRMT2 | Forward: | GCAGTTGGACATGAGAACCGTG |  |
|  | Reverse: | AGGCTCTGGAAGTGGACGCTAA |  |
| PRMT3 | Forward: | CACTGTCTGCTGAAGCCGCATT |  |
|  | Reverse: | GTAGATGACGAGCAGGTTCTGAC |  |
| PRMT4 | Forward: | TTCCAGTCACCACTGTTCGCCA |  |
|  | Reverse: | CCAGGAGGTTACTGGACTTGGA |  |
| PRMT5 | Forward: | CTAGACCGAGTACCAGAAGAGG |  |
|  | Reverse: | CAGCATACAGCTTTATCCGCCG |  |
| PRMT6 | Forward: | TGGCTTTGCCATCTGGTTCCAG |  |
|  | Reverse: | TAGAGGAGCGCCTGTTTCCAGT |  |
| PRMT7 | Forward: | CCACGATGACTACTGCGTATGG |  |
|  | Reverse: | GACGTACCGATCAGTTCTGTCC |  |
| PRMT9 | Forward: | GGACATTGGAGCAGGAACTGGA |  |
|  | Reverse: | GTTTGCTGCCACGACATCACAG |  |
| WDR5 | Forward: | AGTGCCTCAAGACTTTGCCAGC |  |
|  | Reverse: | CGATGAGCGTCTTCAGGCACTG |  |
| ACSL4 | Forward: | GCTATCTCCTCAGACACACCGA |  |
|  | Reverse: | AGGTGCTCCAACTCTGCCAGTA |  |
| LPCAT3 | Forward: | CAGGATACCTGGTCTGCTTCCA |  |
|  | Reverse: | TGAAGAGCCAGTGGATGGTCTG |  |
| GPX4 | Forward: | ACAAGAACGGCTGCGTGGTGAA |  |
|  | Reverse: | GCCACACACTTGTGGAGCTAGA |  |
| NRF2 | Forward: | CACATCCAGTCAGAAACCAGTGG |  |
|  | Reverse: | GGAATGTCTGCGCCAAAAGCTG |  |
| TFRC | Forward: | ATCGGTTGGTGCCACTGAATGG |  |
|  | Reverse: | ACAACAGTGGGCTGGCAGAAAC |  |
| FTH | Forward: | TGAAGCTGCAGAACCAACGAGG |  |
|  | Reverse: | GCACACTCCATTGCATTCAGCC |  |
| FPN | Forward: | GAGACAAGTCCTGAATCTGTGCC |  |
|  | Reverse: | TTCTTGCAGCAACTGTGTCACAG |  |
| GAPPDH | Forward: | GTCTCCTCTGACTTCAACAGCG |  |
|  | Reverse: | ACCACCCTGTTGCTGTAGCCAA |  |

**Supplementary Table 8**. Primer sequences in CUT&RUN-qPCR

| **Gene** | **Sequence 5’-3’** | | **Species** |
| --- | --- | --- | --- |
| ACSL4 - P1 | Forward: | CAGTGTGCTGGATGAAGATGAAAA | Human |
|  | Reverse: | GGCTAAGAGACCGACAATAAAGGA |  |
| ACSL4 - P2 | Forward: | CCAGCGAACTATCTGAGGGC |  |
|  | Reverse: | TCACCATTACGCATCAGGGAA |  |
| ACSL4 - P3 | Forward: | GTTCCCCTCGGGTCATCTTC |  |
|  | Reverse: | AGTGAGCGGGGATCTTAACG |  |
| ACSL4 - P4 | Forward: | GACCCCAGAGTCCATCACAG |  |
|  | Reverse: | GCTATCTATCCGCTCGCCTC |  |
| ACSL4 - P5 | Forward: | GCAAGGAAGATATCGGAAGCG |  |
|  | Reverse: | TCTGTAACTGAGGGCTAGGGA |  |
| Spike in DNA |  | ATAACTCAATGTTGGCCTGTATAGCTTCAGTGATTGCGATTCGCCTGTCTCTGCCTAATCCAAACTCTTTACCCGTCCTTGGGTCCCTGTAGCAGTAATATCCATTGTTTCTTATATAAAGGTTAGGGGGTAAATCCCGGCGCTCATGACTTCGCCTTCTTCCCATTTCTGATCCTCTTCAAAAGGCCACCTGTTACTGGTCGATTTAAGTCAACCTTTACCGCTGATTCGTGGAACAGATACTCTCTTCCATCCTTAACCGGAGGTGGGAATATCCTGCATTCCCGAACCCATCGACGA |  |
