# Supplementary material for: PRMT5 promotes ferroptosis in cisplatin-induced acute kidney injury through WDR5/ACSL4 pathway
Source: Redox Biol. 2026 Oct 1;97:104421. doi: 10.1016/j.redox.2026.104421 (PMC13659685; doi:10.1016/j.redox.2026.104421)
Supplement: Supplementary file 2 [file mmc2.docx]

**Original Western Blot Files**

**Figure 1F**

**Figure 1G**

**Figure 2B**

**Figure 2D**

**Figure 2H**

**Figure 3B**

**Figure 3G**

**Figure 4G**

**Figure 4J**

**Figure 5C**

**Figure 5D**

**Figure 6C**

**Figure 6D**

**Figure 7B**

**Figure 7C**

**Figure 7E**

**Figure 7I**

**Figure 9E**

**Figure 9H**

| **Supplementary Figure 1C** | **Supplementary Figure 1G** |
| --- | --- |
|  |  |

**Supplementary Figure 1I**

**Supplementary Figure 7A**
